# The Tenocyte from Atom to Cell: A Critical Multiscale Synthesis Linking Collagen Chemistry, Tissue Mechanics, Cell Mechanobiology and Mathematical Modelling

**DOI:** 10.3390/ijms27209038

**Published:** 2026-10-12

**Authors:** Mikołaj Stańczak, Bartłomiej Kacprzak, Magdalena Hagner-Derengowska

**Affiliations:** 1 School of Mathematical and Computer Sciences, Heriot-Watt University, Edinburgh EH14 4AS, UK; 2 BK21 MedConcept, 90-349 Łódź, Poland; 3 Sports Research Center, Nicolaus Copernicus University, 87-100 Toruń, Poland

**Keywords:** tenocyte, tendon, collagen, mechanobiology, mechanotransduction, PIEZO1, single-cell transcriptomics, multiscale modelling, viscoelasticity, cross-linking, tendinopathy

## Abstract

The tenocyte builds, maintains, senses and degrades one of the most mechanically demanding extracellular matrices in vertebrates, yet the chemical, physical, biological and mathematical descriptions of this system have developed in largely separate literatures. This review examines the tenocyte–matrix unit as a single causal system spanning eight orders of magnitude in length, organised around five transitions: from collagen chemistry to molecular mechanics; from molecule to fibril and fascicle; from tissue loading to the cellular mechanical stimulus; from stimulus to transcription; and from cellular output back to matrix composition. At each transition, we weigh competing hypotheses, identify where evidence is decisive and where it remains correlative, and state the governing equations. We integrate advances from 2021–2026, including single-cell and spatial transcriptomic atlases of human tendons, the establishment of PIEZO1 as a causal regulator of tendon stiffness, and microstructurally derived constitutive models. We argue that tendon homeostasis is a closed-loop control problem, formalised as a mechanostat with an inverted-U dose–response, and that the recurrent pathologies of tendon-tendinopathy, ageing, diabetic stiffening and fibrotic healing are failures of this loop rather than of any single component. An open-source computational companion (tenocyte-virtual-cell) is introduced, and a prioritised research agenda closes the review.

## 1. Introduction

Tendons transmit and store mechanical energy through a dense, highly aligned matrix of type I collagen that is populated by a sparse and heterogeneous population of fibroblastic cells, collectively termed tenocytes [1,2,3,4]. Three research traditions have studied this tissue, with only partial contact between them. Structural biology and materials science have used tendons as the canonical fibrous composite from which much of the current understanding of collagen packing, fibrillogenesis and fibre mechanics was derived [5,6,7,8,9]. Human physiology has used it as an accessible in vivo system for relating mechanical loading to protein synthesis [4,10,11,12,13]. Cell biology and orthopaedic research have used it to dissect mechanotransduction, matrix turnover and the pathogenesis of a family of common and treatment-resistant disorders [14,15,16,17,18,19].

The consequence of this separation is that the transitions between scales, rather than the scales themselves, are the least understood part of tendon biology. Atomic-scale collagen chemistry is well characterised [20,21,22], as is whole-tendon mechanics in vivo [12,13,23]. The unresolved questions concern how information passes between them. Three examples define the problem addressed in this review. First, the stereoelectronic preference of a 4-hydroxyproline ring for the *exo* pucker is known to raise the triple-helical melting temperature by approximately 30 K [24,25,26], but the route by which such residue-level chemistry propagates to tissue-level relaxation time constants has not been made quantitative. Second, a tissue strain of 5% produces a molecular strain of roughly 0.5% [27,28,29,30]. The mechanisms that accommodate the remaining deformation are known qualitatively, but their relative contributions remain contested [31,32,33,34]. Third, the tenocyte possesses at least six distinct mechanosensory systems [35,36,37,38,39,40], but which of them is rate-limiting for the transcriptional response under physiological loading has not been determined.

A second problem concerns modelling. Mathematical descriptions of tendons are abundant but almost exclusively single-scale and phenomenological. Quasi-linear viscoelasticity reproduces stress relaxation without reference to any molecular structure [41,42], molecular dynamics resolves individual hydrogen bonds without reference to any cell [7,43,44], and remodelling models represent cells while treating mechanics as a scalar input [45,46]. Parameters fitted at one scale are therefore rarely interpretable at another, and predictions do not transfer.

The present review addresses both problems. Its purpose is not to catalogue tendon biology level by level, which has been done well elsewhere [3,4,14,16,47,48], but to examine the five causal transitions that connect the scales, to evaluate the strength of the evidence at each, and to specify the mathematical objects that would be required to couple them. A central argument is that the tenocyte does not merely inhabit the structural hierarchy but produces and regulates it: it secretes procollagen [49,50], templates fibril geometry [51,52], installs cross-links [53,54], degrades the matrix through metalloproteinases [55,56], and does so under regulation by the mechanical state of the structure it is building [35,57,58]. The hierarchy is therefore a closed loop, and we contend that the most persistent puzzles in tendon pathology the dual aetiology of tendinopathy, the age-related loss of fatigue resistance, the persistent inferiority of healed tissue follow from the dynamics of this loop rather than from the failure of any single component.

The review is restricted to the tendon midsubstance of mature mammals. The enthesis and myotendinous junction, which involve mineralisation gradients and distinct developmental programmes [59,60,61], are discussed only where they inform midsubstance mechanobiology. Clinical management of tendinopathy is covered comprehensively in recent primers [18,19,62] and is not repeated here.

## 2. Approach and Organisation

### 2.1. Analytical Method

**Figure 1:**
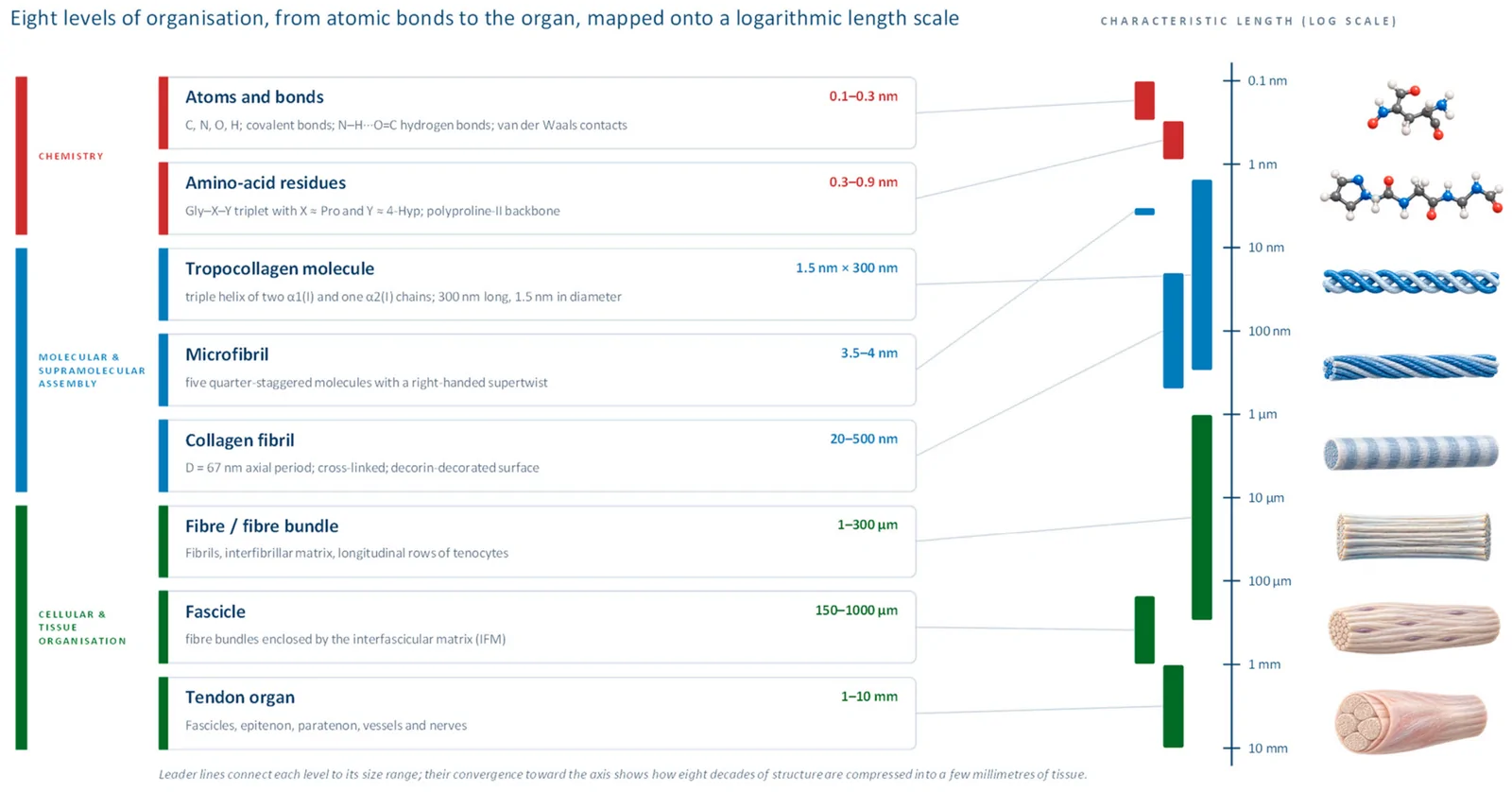
The structural hierarchy of tendons from atomic bonds to the organ, with characteristic dimensions. The colour bands indicate the domains in which chemistry (red), molecular and supramolecular assembly (blue), and cellular and tissue organisation (green) dominate the description. IFM, interfascicular matrix.

**Figure 2:**
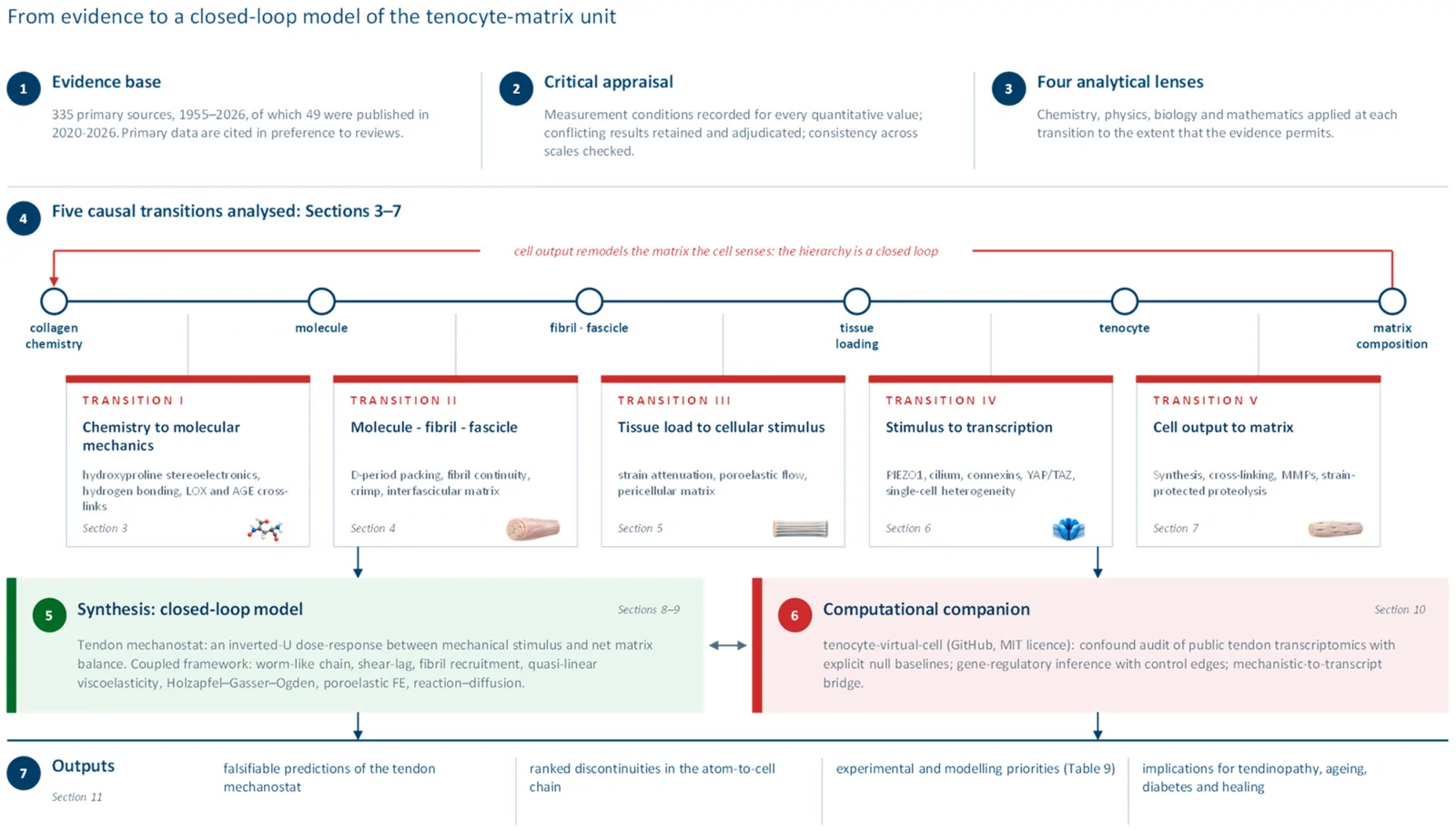
Schematic of the analytical approach adopted in this review. The evidence base is subjected to critical appraisal and examined through four analytical lenses at each of five causal transitions (Section 3, Section 4, Section 5, Section 6 and Section 7). The synthesis (Section 8 and Section 9) yields a closed-loop model of tendon homeostasis and a coupled mathematical framework complemented by an open-source computational companion (Section 10). Outputs are falsifiable predictions and a prioritised research agenda (Section 11).

Figure 1 summarises the structural hierarchy that provides the spatial coordinate system for this review, and Figure 2 summarises the analytical approach. The evidence base comprises primary literature from 1955 to September 2026. Approximately 15% of the 335 cited sources were published in 2020–2026, reflecting the rapid growth of single-cell transcriptomic and mechanosensor studies in this period. Where quantitative values are reported, the measurement modality and the principal conditions that influence the value (species, hydration, gauge length, strain rate) are stated, because differences in these conditions account for most of the apparent disagreements in the literature [63,64,65,66,67]. Where studies conflict on mechanism rather than magnitude, both positions are presented with the evidence for each and the authors’ assessment is stated explicitly.

Four analytical lenses chemistry, physics, biology and mathematics are applied at each transition to the extent that evidence permits. We do not claim, and it would not be credible to claim, that all four are equally developed at every scale: the mathematical description of the cellular compartment, for instance, is far less mature than that of the collagen fibril, and this asymmetry is itself one of the findings of the review (Section 8.8).

### 2.2. Composition and Functional Classes

By wet weight, mature tendon is 55–70% water. Of the dry mass, 60–85% is collagen, of which more than 95% is type I in the midsubstance [4,47,68,69]. Proteoglycans account for less than 5% and elastin for 1–2% of dry weight [70,71,72,73]. These proportions are a poor guide to functional importance. Elastin, though a minor constituent, is concentrated in the interfascicular matrix and around cells, and its enzymatic removal selectively degrades interfascicular mechanics [72,73,74]. Decorin and biglycan are required to maintain the mechanical properties of a mature tendon [75,76]. Lysyl oxidase-derived cross-links constitute a negligible mass fraction, but their inhibition abolishes ordered fibrillogenesis [54,77]. Water, the majority constituent, is often treated as mechanically inert, even though it governs triple-helical stability [78,79], mediates the intra- and interfibrillar swelling that underlies stress relaxation [80], and constitutes the fluid phase of the poroelastic response [81,82].

Tendons are functionally specialised, and the specialisation is implemented at a specific hierarchical level. Positional tendons transmit force with minimal energy storage. Energy-storing tendons such as the human Achilles and the equine superficial digital flexor undergo large cyclic strains and return stored elastic energy with high efficiency [83,84,85]. Fascicles from the two classes have broadly similar properties. The functional difference resides principally in the interfascicular matrix, which in energy-storing tendons permits greater fascicle sliding and more complete recovery [86,87,88]. This finding, established in equine tendons and confirmed in human Achilles tendons [88], relocates the determinants of both energy-storing function and rupture risk to a compartment that until recently received little attention and that still lacks a validated constitutive description (Section 4.6).

### 2.3. Time Scales

The hierarchy of length scales (Table 1) is matched by a hierarchy of time scales spanning more than fifteen decades: femtosecond bond vibration and hydrogen-bond exchange [78,89]; nanosecond-to-microsecond conformational fluctuation of the tropocollagen molecule [7,43]; millisecond-to-second ion channel gating and calcium transients [38,90]; second-to-minute poroelastic fluid redistribution and intercellular calcium waves [30,91,92]; minute-to-hour transcriptional responses [40,93,94]; hour-to-day protein synthesis and protease induction [55,58]; and year-to-decade matrix turnover and advanced glycation end-product accumulation [95,96,97,98]. Adjacent scales are strongly coupled while distant scales are not, so that a tendon acts as a low-pass filter on mechanical history. This explains why acute overload and chronic overuse produce different pathologies in the same tissue [57,99,100]. It constrains any model that attempts to couple mechanics to biology; the coupling must be integrative rather than instantaneous.

## 3. Transition I: From Collagen Chemistry to Molecular Mechanics

### 3.1. The Sequence Constraint and Its Mechanical Meaning

Type I collagen is a heterotrimer of two α1(I) chains and one α2(I) chain, each contributing a triple-helical domain of approximately 1014 residues obeying a near-inviolable (Gly-X-Y)_n_ repeat [111,112]. The constraint is steric: the three chains are staggered by one residue and wound about a common axis, placing one residue of each triplet against the axis in a space that accommodates only a hydrogen atom [113,114,115]. Glycine substitution introduces a side chain into this space, kinks the helix and impairs propagation. Because the defective chain co-assembles with normal chains, the mutations are dominant-negative, which is the molecular basis of osteogenesis imperfecta and the classical Ehlers–Danlos syndromes [111,116]. Proline is enriched at X (≈28%) and 4-hydroxyproline at Y (≈38% of Y positions), and both imino acids pre-organise the chain toward the polyproline-II conformation required for assembly, lowering the entropic cost of folding [21,117,118]. Residue-based propensity scales derived from host–guest peptides predict melting temperature from sequence with useful accuracy [117,118].

### 3.2. Hydrogen Bonding and Structured Water

The helix is held axially by a single conserved class of direct interchain hydrogen bond, from the glycine amide N–H to the carbonyl of the X-position residue of the adjacent chain one bond per triplet per chain pair [20,115]. Crystallography of collagen-like peptides revealed an extensive second shell of water-mediated hydrogen bonds bridging hydroxyproline hydroxyls, backbone carbonyls and neighbouring chains [20,78]. Osmotic-stress force measurements between triple helices demonstrated a temperature-dependent exponential repulsion attributable to structured interfacial water [89], and sugars that disrupt hydrogen-bonded water bridges inhibit fibrillogenesis [119]. The mechanical corollary is that collagen stiffness depends on its thermodynamic environment; hydrated and dehydrated fibril moduli differ by roughly an order of magnitude [65,67,120], a difference that is physical rather than artefactual and that must be respected when comparing studies.

### 3.3. Prolyl 4-Hydroxylation: A Resolved Mechanistic Debate

Prolyl 4-hydroxylation is the best-documented chemistry-to-mechanics link in the hierarchy and illustrates the standard of causal continuity to which the remainder of the review is held. The synthetic peptide (Pro-Pro-Gly)_10_ melts near 30 °C, (Pro-Hyp-Gly)_10_ melts near 60 °C [24], and non-hydroxylated collagen produced under ascorbate deprivation is unstable at body temperature and is not secreted normally [121,122].

The mechanism was disputed for two decades. The original interpretation attributed the stabilisation to additional water-mediated hydrogen bonds involving the 4-hydroxyl group [20,78]. Raines and colleagues showed that this explanation is insufficient: 4-fluoroproline, which cannot donate a hydrogen bond but is more electron-withdrawing than the hydroxyl, stabilises the helix more strongly than hydroxyproline [25,123]. The dominant contribution is stereoelectronic. The electronegative 4R substituent favours the *exo* ring pucker, which favours the *trans* amide isomer and the backbone dihedral angles of the polyproline-II conformation [26,101,124]. Hybrid density-functional calculations quantified the electronic origin of this preference [101,124]. In our assessment, the debate is settled in favour of the stereoelectronic mechanism at the level of the isolated helix. Hydration remains decisive for interhelical packing and fibril-scale mechanics [65,89], so the two explanations operate at different levels of the hierarchy rather than competing at the same one.

The enzymology completes the chain. Prolyl 4-hydroxylase is a non-haem Fe^2+^, 2-oxoglutarate-dependent dioxygenase that consumes O_2_ and uses ascorbate to rescue the enzyme from uncoupled cycles in which the iron is oxidised [125,126,127,128]. Scurvy is therefore a disease of stereoelectronics. Ascorbate deficiency inactivates the enzyme, the *endo* pucker is permitted, the helix melts at physiological temperature, procollagen is degraded intracellularly, fibrils do not form and connective tissue fails [111,122,128]. The oxygen dependence has an under-examined consequence specific to tendons. The tissue is hypovascular and relatively hypoxic [69,129], so that collagen’s post-translational quality control is coupled to metabolic state through the same dioxygenase family that mediates hypoxia signalling.

### 3.4. Lysine Modification, Glycosylation and Enzymatic Cross-Linking

Lysyl hydroxylases (LH1–3) hydroxylate specific lysines, and the pattern of hydroxylation at telopeptide versus helical sites determines which cross-link chemistry can subsequently form [130,131,132]. Hydroxylysine may be further galactosylated or glucosylgalactosylated by the bifunctional LH3, and site-specific glycosylation differs between tissues and modulates fibril diameter [133,134]. Table 2 summarises the residue-level chemistry.

Lysyl oxidase (LOX), a copper-dependent amine oxidase, oxidatively deaminates telopeptide lysine or hydroxylysine to aldehydes that condense spontaneously with helical lysine or hydroxylysine on adjacent molecules, forming divalent immature cross-links that mature over months to years into trivalent pyridinolines [53,95,132,135,136]. Because LOX targets telopeptides, enzymatic cross-links are geometrically defined within the D-period gap region rather than randomly distributed. LOX inhibition abolishes ordered fibrillogenesis in tendon-like constructs [54], and LOX-mediated cross-linking correlates quantitatively with the acquisition of mechanical properties during embryonic tendon development [77,137]. Molecular simulation of the C-terminal cross-link domain shows the cross-link to be a load-bearing element with its own failure mechanics [138], and single-fibril fracture experiments confirm that natural cross-links dominate fibril strength [105].

**Table 2:** Post-translational chemistry of type I collagen relevant to tendon mechanics.

Modification	Enzyme (Gene)	Cofactors/Cosubstrates	Structural Consequence	Mechanical Consequence	Refs.
Proline 4-hydroxylation	Prolyl 4-hydroxylase (*P4HA1–3*)	Fe^2+^, 2-oxoglutarate, O_2_, ascorbate	*Exo* pucker; *trans* amide; PPII pre-organisation	ΔT_m_ ≈ +30 K; permits secretion and fibrillogenesis	[24,25,26,125,127]
Lysine hydroxylation	Lysyl hydroxylases (*PLOD1–3*)	Fe^2+^, 2-oxoglutarate, O_2_, ascorbate	Generates hydroxylysine; sets cross-link route	Determines mature cross-link chemistry	[130,131,132]
Glycosylation	LH3 (*PLOD3*), GLT25D1/2	UDP-sugars, Mn^2+^	Bulky adducts at fibril surface	Modulates fibril diameter and packing	[133,134]
Oxidative deamination	Lysyl oxidase (*LOX*, *LOXL1–4*)	Cu^2+^, lysine tyrosylquinone, O_2_	Allysine; initiates cross-linking	Required for fibrillogenesis and modulus	[53,54,77,136]
Non-enzymatic glycation	None (Maillard chemistry)	Reducing sugars	Glucosepane, pentosidine	Restricts sliding; embrittles	[96,139,140,141,142]
Propeptide cleavage	ADAMTS2; BMP1/mTLD	Zn^2+^	Converts procollagen to collagen	Prerequisite for assembly	[49,50,112]

PPII, polyproline-II.

### 3.5. Non-Enzymatic Glycation: The Evidence Re-Examined

Reducing sugars react with helical lysine and arginine through Maillard chemistry, forming Amadori products that rearrange over years into advanced glycation end-products (AGEs), of which pentosidine is the best-characterised fluorescent marker and glucosepane the most abundant [139,140,143]. Because glycation is non-enzymatic, it accumulates on helical residues throughout the molecule, at a rate set by glucose concentration and protein residence time. Tendon collagen is maximally vulnerable: radiocarbon bomb-pulse dating shows essentially no replacement of the collagen core of healthy adult Achilles tendons after skeletal maturity [97], and aspartic acid racemisation confirms the accumulation of long-lived, damaged collagen with age [144].

The mechanical consequences were characterised by Snedeker, Gautieri and colleagues. Ribose-induced glycation diminishes fibre sliding in fascicles [141] and reduces molecular sliding within fibrils without necessarily altering small-strain stiffness [142]. These effects are reproduced in molecular dynamics simulations that incorporate the specific chemistry of glucosepane and pentosidine bridges [96,145]. Single-fibril tests show that maturation and glycation alter fibril mechanics measurably [146]. Mesoscale simulations of cross-link density predict a non-monotonic relation with toughness. Stiffness and strength rise with density, but beyond an optimum, suppressed intermolecular sliding makes the fibril brittle [104]. A 2026 extension of the ColBuilder framework now permits insertion of glucosepane, pentosidine and other non-enzymatic cross-links into atomistic fibril models, which should allow this prediction to be tested with explicit chemistry [147].

Two recent findings temper the prevailing narrative. In human Achilles tendons, Zellers and colleagues found that mechanical behaviour correlated more strongly with collagen disorganisation than with AGE content [148], and Vaidya, Lake and Zellers argued that diabetic tendon dysfunction extends beyond cross-linking to nanoscale changes in discrete plasticity and in the D-period [149]. A multiscale fibril-reinforced model of the knee tendon nonetheless predicts substantial stiffness changes from plausible AGE densities [150]. Our reading of this evidence is that the AGE mechanism is real and well supported at the molecular and fibril levels, but that its contribution to tissue-level pathology in humans has probably been overstated relative to collagen disorganisation and to cellular effects of the glycated environment, including RAGE signalling [151,152]. Table 3 contrasts the two cross-linking systems.

### 3.6. Molecular Mechanics and the Role of Simulation

A tropocollagen molecule is a semiflexible polymer with an aspect ratio of approximately 200 and a persistence length of 10–20 nm [102,153,154]. At low force, it behaves as a worm-like chain whose restoring force is entropic:

$$F\left(x\right)=\frac{k_{B}T}{L_{p}}\left[\frac{1}{4{\left(1-x/L_{c}\right)}^{2}}-\frac{1}{4}+\frac{x}{L_{c}}\right]$$

where x is the end-to-end extension, L_c_ the contour length and L_p_ the persistence length. Once nearly straight, further extension requires deformation of backbone bonds, unwinding of the supercoil and disruption of interchain hydrogen bonds, and the response becomes enthalpic with a tangent modulus of 3–9 GPa depending on hydration and strain rate [27,28,44,103,155,156]. Deformation rate controls both the apparent modulus and the unfolding pathway of single molecules [103], which is one origin of the strain-rate sensitivity observed at every higher level.

Molecular dynamics is the instrument that renders this transition quantitative, because the force-field parameters are the chemistry of Section 3.1, Section 3.2, Section 3.3, Section 3.4 and Section 3.5. Dihedral terms encode ring-pucker preferences altered by hydroxylation, electrostatic terms encode the hydrogen-bond network, and explicit water reproduces the hydration shell. Gautieri and colleagues built a full atomistic microfibril from the in situ X-ray structure and obtained a stiffness of approximately 300 MPa at low strain rising to 1.2 GPa above 10% strain, with explicit identification of the responsible deformation mechanisms [43]. This remains the clearest demonstration that mesoscale mechanics can be derived from atomic chemistry. Its limitation is temporal: all-atom simulations reach microseconds, whereas the phenomena of physiological interest occupy seconds to hours, so that coarse-grained representations with fitted effective potentials are required to bridge the gap, at the cost of chemical resolution [7,104].

### 3.7. Critical Appraisal of Transition I

The chemistry-to-molecule transition is the most secure link in the chain. Its principal weakness is not evidential but quantitative: the propagation of residue-level thermodynamic parameters to tissue-level viscoelastic constants has never been carried through explicitly, and the coarse-grained models that would be required to do so are parameterised against atomistic data in ways that are not always transparent. Two specific gaps merit emphasis. First, the optimum cross-link density predicted by mesoscale simulation [104] has not been tested experimentally in tendon by systematic variation of LOX activity combined with fibril-level fracture testing. Second, the balance between AGE-mediated embrittlement and collagen disorganisation as determinants of human tendon failure remains unresolved [148,149], and the two mechanisms are not independent, because sliding restriction alters the strain distribution that drives disorganisation.

## 4. Transition II: From Molecule to Fibril and Fascicle

### 4.1. Quarter-Stagger Packing and the D-Period

**Figure 3:**
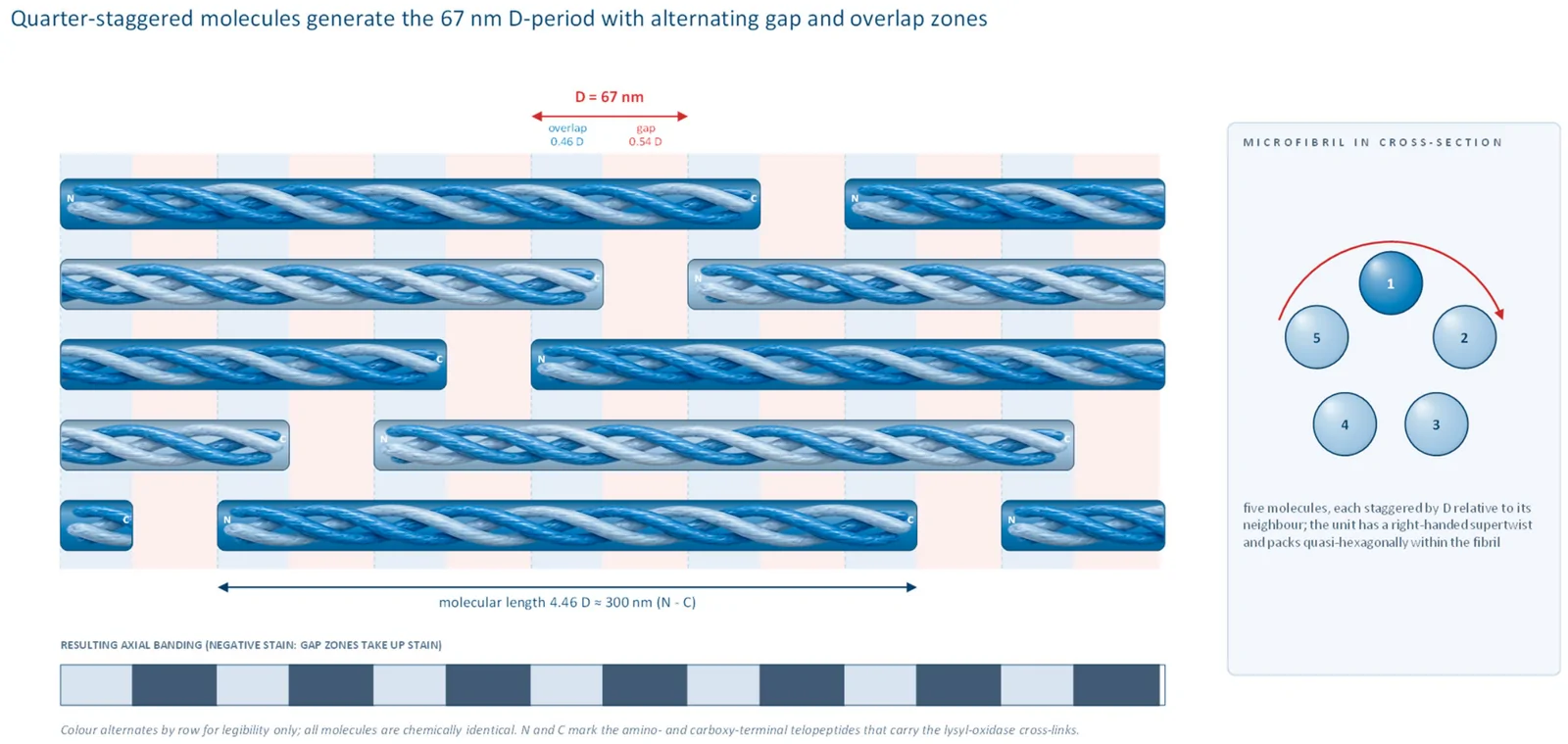
Quarter-staggered axial packing of tropocollagen molecules generating the 67 nm D-period. Molecular length ≈ 4.46 D; gap zone ≈ 0.54 D; overlap zone ≈ 0.46 D.

Tropocollagen molecules pack in axial register with a stagger of approximately one quarter of their length and a gap between the C-terminus of one molecule and the N-terminus of the next, producing alternating gap and overlap zones with a repeat distance (D-period) of approximately 67 nm in hydrated tendon [8,9,157]. The molecular length is close to 4.46 D, the overlap zone occupies approximately 0.46 D and the gap zone approximately 0.54 D (Figure 3). The three-dimensional structure was resolved in situ by synchrotron X-ray diffraction: the fundamental packing unit is a right-handed supertwisted microfibril of five staggered molecules, organised quasi-hexagonally and interdigitating so that the fibril is not a bundle of discrete cables [9,158,159,160]. This architecture, together with local helical conformation, governs proteolytic susceptibility. The collagenase cleavage site is accessible in specific structural contexts and shielded in others [161], a structure-to-turnover link that becomes central in Section 7.

Under tensile load, the D-period increases, and the increase partitions unequally between gap and overlap zones, indicating a more compliant gap region [29,162,163]. Fibril strain measured from the D-period is systematically smaller than applied tissue strain, which is the primary experimental evidence for strain attenuation across the hierarchy (Section 5.2).

### 4.2. Fibril Mechanics: Reconciling a Two-Decade Spread of Values

Direct testing of individual fibrils by MEMS devices, AFM tension, and nanoindentation has produced moduli spanning two orders of magnitude [63,64,65,66,105,120,164,165,166,167]. The dispersion is now understood to arise principally from hydration (dehydration raises the value by roughly an order of magnitude [65,120]), loading mode (tension versus indentation), strain level and rate, and cross-link maturity, as synthesised in a recent critical review of single-fibril mechanics [67]. For hydrated fibrils in tension, the defensible range is 0.4–2.9 GPa [63,120,164,167]. Recent work using collagen-hybridising peptides has shown that molecular denaturation within fibrils begins at the yield point of the fibril stress–strain curve in both positional and energy-storing tendons, with greater denaturation in positional fibrils despite their lower yield stress [168]. Fibrils from the two tendon classes also differ in their strain-stiffening and their structural response to fatigue [169]. These results establish that the fibril, not the whole tissue, is the level at which overload is converted into irreversible molecular damage, and they provide a molecular readout for the yield point that models must reproduce.

### 4.3. Fibril Continuity: A Controversy That Determines the Load-Transfer Model

Whether fibrils in mature tendons are continuous or discontinuous determines whether load enters each fibril at its ends or through interfibrillar shear, and therefore which constitutive model is appropriate. The evidence is genuinely divided and deserves a careful statement.

The evidence for discontinuity is predominantly mechanical. Szczesny and Elliott demonstrated that a shear-lag model reproduces both macroscale fascicle mechanics and microscale fibril strains, which requires load transfer through the interfibrillar matrix [31], and that this matrix must be modelled as plastic rather than elastic to capture post-yield behaviour [32,170]. Peterson and Szczesny subsequently showed that fibril strain equals tissue strain only at gauge lengths of 5 mm, whereas at 20 mm and above fibril strains fall below tissue strains, consistent with fibrils that are 5–10 mm long [34]. Developmental studies show fibrils growing from short segments that fuse with maturation [51,171]; early estimates of mean fibril length in rat tail tendons were finite [172].

Evidence for continuity is predominantly structural. Svensson and colleagues, using serial block-face scanning electron microscopy, observed a single fibril end after tracking 67.5 mm of cumulative fibril length in an adult human tendon, and confirmed the continuity of individual fibrils across the full length of a mouse stapedius tendon [33]. Hijazi, Singfield and Veres showed that under static overload, tendon rupture proceeds by fibril failure rather than uncontrolled interfibrillar slippage, implying fibrils long enough to be mechanically continuous [173]. Extensive earlier electron microscopy of mature tendons and ligaments detected no fibril ends [174].

The two bodies of evidence are reconcilable if fibrils are very long but finite, on the order of millimetres, so that they behave as continuous over short gauge lengths and as discontinuous over physiological ones [33,34]. This is our working position, and it has a specific consequence: shear-lag load transfer (Section 8.5) is the appropriate model for fascicle-scale mechanics at physiological gauge lengths, but the interfibrillar shear parameter it requires must be interpreted as a property averaged over fibril lengths that vary with age and development. Resolution requires volume electron microscopy over millimetre lengths in mature human tendons, which is technically demanding but now feasible.

### 4.4. Regulation of Fibril Assembly: Proteoglycans and Glycoproteins

Fibril diameter distribution in mature tendons is broad and often bimodal (20 to >500 nm) [174,175,176], and is regulated by nucleating minor collagens (types V and XI) and by surface-binding small leucine-rich proteoglycans (SLRPs) [50,175,177,178]. Decorin constitutes approximately 80% of tendon proteoglycan core protein [71]. Decorin-null mice display irregular fibrils [75], and inducible deletion of decorin and biglycan in mature tendons impairs fibril structure, fibre realignment and mechanical properties [76], establishing that SLRPs are required for maintenance and not only for development. Fibromodulin and lumican act at distinct developmental stages [178,179,180].

The proposal that decorin glycosaminoglycan (GAG) chains bridge adjacent fibrils and transmit load, formalised computationally by Redaelli and colleagues [181], was tested directly and largely refuted. Enzymatic GAG depletion produced no significant change in quasi-static or dynamic fascicle mechanics [182,183] and only a modest regional dependence [184]. Similar results were obtained in human ligaments [185]. A modified shear-lag analysis showed that GAGs could contribute to load transfer only at densities and stiffnesses not physiologically realised [186]. The current consensus, which we share, is that interfibrillar load transfer is mediated by direct fibril–fibril interaction and the bulk properties of the interfibrillar matrix, and that decorin acts on mechanics indirectly, through the regulation of fibril diameter, spacing and surface properties [31,32,76]. This episode is instructive: a structurally plausible hypothesis survived for a decade until mechanical testing with appropriate controls adjudicated it.

Among glycoproteins, cartilage oligomeric matrix protein acts catalytically in fibrillogenesis and is enriched in high-load tendons [187,188,189]. Tenascin-C is strongly and directly mechanoregulated through Rho/ROCK signalling and modulates cell adhesion [190,191,192]. The elastic fibre system (elastin, fibrillin-1/2) is concentrated in the interfascicular matrix and around cells, where it provides recoverable extensibility and sequesters latent TGF-β [72,73,74,193,194]. Table 4 summarises the non-collagenous inventory.

### 4.5. Crimp

Unloaded tendon fibres exhibit a planar waveform with crimp angles ranging from a few degrees to approximately 20° [1,2,201,202]. Kastelic and colleagues produced the first structural-mechanical model of crimp [2]. Optical measurements confirmed progressive recruitment of crimped fibres with applied strain [203]. Franchi and colleagues showed that crimp exists at multiple levels, including a fibrillar crimp corresponding to left-handed helical arrangements of fibrils [202,204]. Because individual elements straighten at different strains, they are recruited progressively, producing the concave-up toe region formalised in Section 8.3. Crimp is actively generated: tenocyte contraction induces crimp in engineered tendon-like tissue [205], a direct instance of the cell producing the structure that subsequently determines the strain it experiences.

### 4.6. The Interfascicular Matrix

The interfascicular matrix (IFM) is compositionally distinct from the fascicular matrix, enriched in elastin, lubricin, proteoglycans and fibrillin microfibrils, and is more cellular and vascular than the fascicular matrix [72,73,193]. It permits fascicles to slide and recover, which is the mechanism by which energy-storing tendons achieve their extensibility and efficient recoil [86,87]. IFM properties differ systematically between functional classes and deteriorate with age far more than fascicular properties [73,87,206]. The human Achilles tendon shows the same structure–function specialisation of the IFM [88]. Postnatal mechanical loading drives tissue adaptation primarily through the non-collagenous matrix rather than the collagen [198]. Recent single-cell work has redefined the IFM as an active biological compartment rather than a passive lubricating layer: it houses nine of the eleven cell clusters identified in equine superficial digital flexor tendons, including interfascicular tenocytes and mural cells that are preferentially affected by ageing [207], and its basement membrane provides a vascular niche for CD146^+^ progenitor subpopulations recruited during injury through laminin-α4 [208,209].

We regard the IFM as the most consequential and least characterised compartment of tendons. It is the probable locus of energy-storing tendon rupture, the compartment most affected by ageing, and the site of the progenitor niche, yet no accepted strain-energy function or viscoelastic formulation exists specifically for it. Section 11 assigns it first priority for constitutive development.

## 5. Transition III: From Tissue Loading to the Cellular Mechanical Stimulus

### 5.1. The Stress–Strain Response and Its Structural Correlates

**Figure 4:**
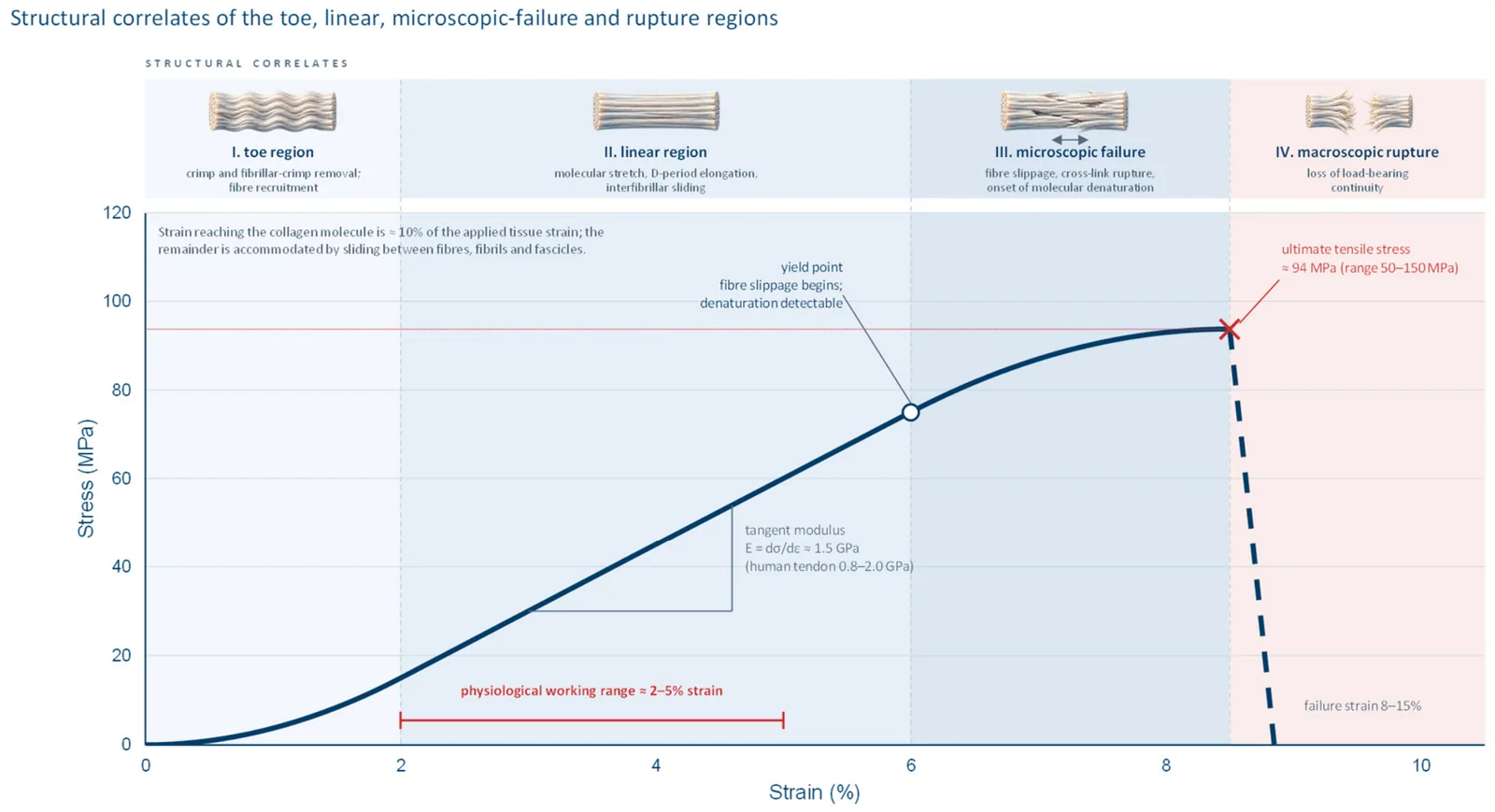
Tensile stress–strain response of tendon with structural correlates. Upper strip: schematic fibre architecture in each region (I, crimped fibres; II, straightened fibres under uniform load; III, discontinuous fibrils sliding past one another; IV, loss of continuity). Open circle, yield point at which fibre slippage begins and molecular denaturation becomes detectable [168]; cross, ultimate tensile stress; bracket, physiological working range. The curve is a schematic constitutive sketch (quadratic toe region to 2% strain, linear region with a tangent modulus of 1.5 GPa, smooth loss of stiffness beyond yield and abrupt rupture); region boundaries and magnitudes vary with tendon type, species, hydration and strain rate [12,23,211].

Uniaxial testing of tendon yields a three-region response (Figure 4). In the toe region (≈0–2% strain), stiffness rises as crimp is removed and fibres are progressively recruited [1,2,201,203]. In the linear region (≈2–5%), the tangent modulus is approximately constant and deformation proceeds by molecular stretch, D-period elongation, intermolecular slip and interfibrillar sliding [29,30,31,163]; the modulus of human tendon in this region is 0.8–2.0 GPa, with energy-storing tendons at the lower end [12,13,23,210]. Yield and failure (above ≈4–8%) proceed through fibre slippage, cross-link rupture and non-recoverable sliding, with molecular denaturation beginning at the fibril yield point [168,211,212]. Damage accumulates well below the ultimate failure strain under repeated sub-failure loading [206,212,213].

### 5.2. Strain Attenuation Across the Hierarchy

**Figure 5:**
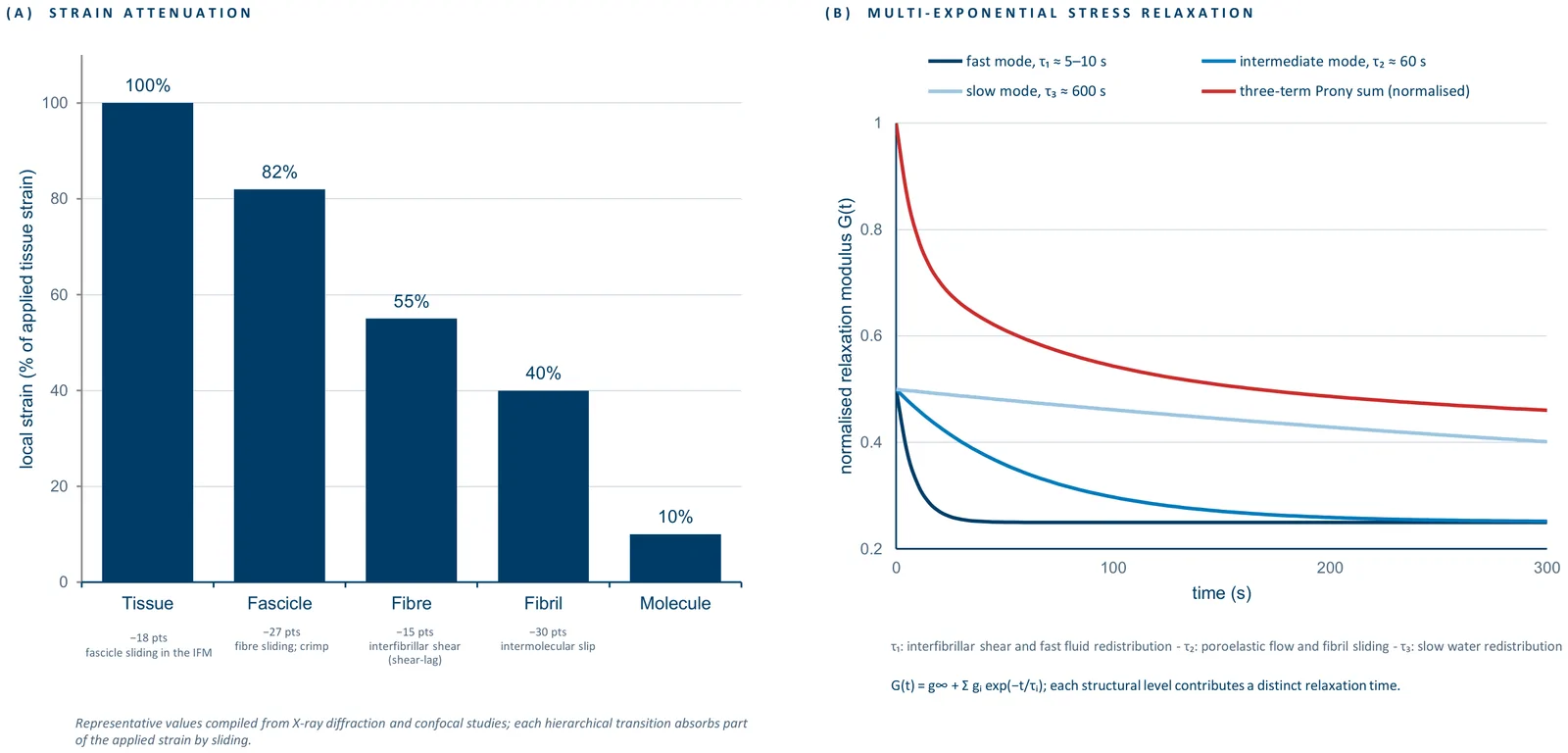
(**A**) Attenuation of local strain relative to applied tissue strain across hierarchical levels (representative values compiled from X-ray diffraction and confocal studies [27,29,30,214]). (**B**) Multi-exponential stress relaxation: individual relaxation modes with fast, intermediate and slow time constants and their normalised Prony sum, illustrating that each structural level contributes a distinct relaxation time.

The most important quantitative fact for the atom-to-cell programme is that strain is attenuated at every hierarchical transition. X-ray diffraction shows molecular strain approximately an order of magnitude below tissue strain [27,28]; synchrotron small-angle scattering quantifies fibril strain as a fraction of tissue strain [29,163]; confocal microscopy with photobleached fiducials shows substantial fibre and fascicle sliding [80,214]; in situ multi-level analysis resolves the partition among molecular, fibrillar and interfibrillar contributions [30]; and the same attenuation, sliding and reorganisation occur under shear and compression [108]. The consensus picture (Figure 5A) is that the strain reaching the collagen molecule is roughly a tenth of that applied to the tissue, the remainder being absorbed by sliding mechanisms that dissipate energy and confer toughness.

Two consequences follow. First, molecular-level stresses computed under an affine assumption overestimate the true values by roughly an order of magnitude, which matters for any argument about mechanically modulated proteolysis or molecular damage. Second, anything that suppresses, sliding most notably AGE cross-linking, removes the principal energy-absorbing mechanism and embrittles the tissue [96,141,142].

### 5.3. Viscoelasticity and Poroelasticity

Tendons exhibit stress relaxation, creep, hysteresis, strain-rate-dependent stiffness and preconditioning [41,215,216,217,218]. At least four physically distinct dissipation mechanisms operate on different time scales: poroelastic interstitial fluid flow with characteristic time τ_p_ ~ L^2^/(H_A_k) [81,82,219]; viscous interfibrillar and interfascicular shear [31,32,87]; intrafibrillar molecular slip resisted by cross-links [104,163]; and nanoscale water redistribution between and within fibrils, identified by Screen and colleagues as a mechanism distinct from bulk flow [80]. Relaxation is accordingly multi-exponential, requiring three or more Prony terms with time constants from seconds to many minutes (Figure 5B) [91,220]. Poisson’s ratios well above 0.5 indicate volume loss arising from fluid exudation and the helical fibre architecture [221,222], and the resulting interstitial flow generates shear stress at the cell membrane that scales with strain rate rather than strain amplitude [109].

### 5.4. What the Tenocyte Experiences

**Figure 6:**
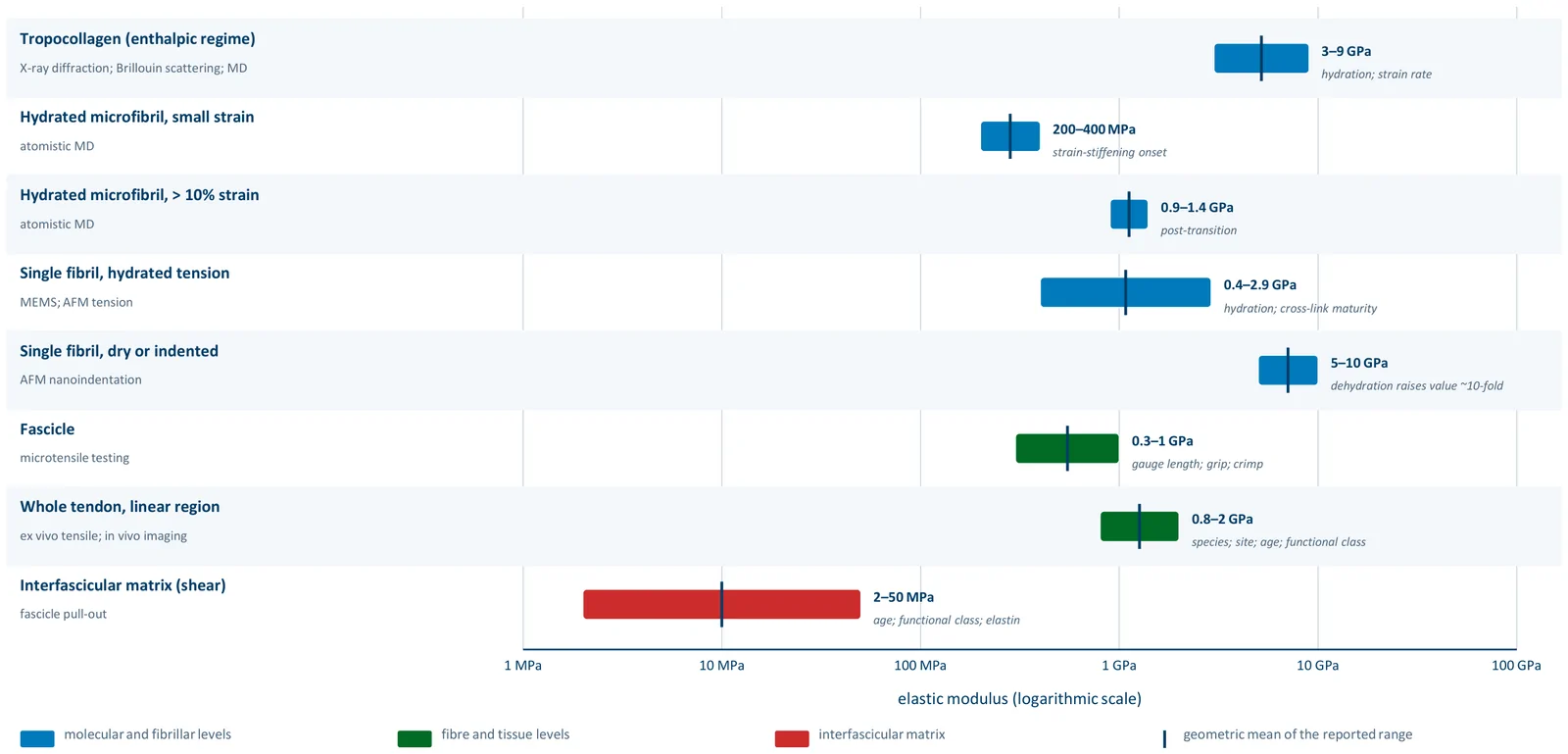
Elastic modulus across the tendon hierarchy. Bars show the range of values reported under the conditions listed in Table 5; red marks indicate geometric means.

Converting tissue loading into the cellular mechanical environment requires accounting for strain attenuation through the hierarchy; local strain modulation by the pericellular matrix, which is softer than the fibrillar matrix and rich in collagen VI, fibronectin and elastic fibres [72,108,193,199,223]; fluid shear proportional to strain rate [109]; hydrostatic pressure, which in compressed regions drives fibrocartilaginous differentiation [224,225]; and osmotic effects of fixed charge density [70,71]. These are distinct physical stimuli capable of activating different sensors, which explains why in vitro studies employing a single modality, usually uniaxial substrate stretch, often fail to reproduce in vivo behaviour, and why explant and three-dimensional systems in which the full stimulus set is present have become the preferred platforms [226,227]. Table 5 collates mechanical property values across the hierarchy. Figure 6 shows the corresponding modulus ranges.

### 5.5. Critical Appraisal of Transitions II and III

Strain attenuation is robust because it has been demonstrated by independent methods, but the quantitative partition among mechanisms rests heavily on rat tail tendon fascicles, a positional tissue, and on equine tendons. Comparable multiscale data for human energy-storing tendons are sparse. The poroelastic description is theoretically mature but its parameters permeability, aggregate modulus and drainage path are rarely measured in tendons and are often borrowed from cartilage. Cell-level finite element models depend on pericellular matrix properties that have not been measured in situ. The fibril continuity question remains open, and until it is resolved the interfibrillar shear parameter of shear-lag models cannot be given an unambiguous structural interpretation. These limitations do not undermine the qualitative picture, but they mean that the cellular mechanical environment is at present inferred rather than measured, which is a serious weakness for a field whose central claim is that cells respond to that environment.

## 6. Transition IV: From Cellular Mechanical Stimulus to Transcription

### 6.1. The Cell Population Is Not Homogeneous

Any account of how “the tenocyte” converts a mechanical stimulus into a transcriptional response must first confront the finding that the resident cell population of tendons is heterogeneous, and that this heterogeneity is spatially organised. Until 2020, the tenocyte was defined by location and morphology: an elongated cell aligned with the fibre axis, connected to its neighbours by gap-junction-coupled processes in a three-dimensional network [224,228]. Single-cell and single-nucleus transcriptomics have since resolved this population into multiple subtypes in mouse [229], horse [207] and human [230,231,232,233] tendons (Table 6).

Three features of these atlases are relevant to mechanobiology. First, fibroblast subpopulations partition by anatomical compartment. In equine energy-storing tendons, one tenocyte cluster maps to the fascicular matrix while nine clusters map to the interfascicular matrix, and the interfascicular populations are those disproportionately affected by ageing, with signatures of senescence, dysregulated proteostasis and inflammation [207]. In human Achilles tendons, fibroblast specialisation follows microanatomy from enthesis through midbody to the muscle–tendon junction [233]. Second, disease reorganises the population. In human tendinopathy, Fu and colleagues resolved ten tenocyte subpopulations and used spatial transcriptomics to order the progression from inflammatory infiltration through chondrogenic transformation to endochondral ossification [231], and combined single-cell and spatial profiling has implicated dysregulated immune homeostasis as an early feature of disease [234]. Third, mechanical responsiveness differs between subpopulations. Single-cell profiling of human tendon progenitor cells under stretch showed that cells from diseased tendons mount a distinct response involving altered mitochondrial biogenesis [235].

These findings have a direct methodological consequence for the present review. Essentially all quantitative mechanobiological data on tendon cells load-dependent MMP-1 suppression [58,236], collagen synthesis under cyclic strain [237,238], and gap-junction modulation [92,239] were obtained on bulk populations or on unfractionated primary cultures, and therefore represent averages over subpopulations that may differ in mechanosensitivity, matrix output and fate. Models that treat the cellular compartment as homogeneous, which is the case for every published model of tendon mechanobiology including the framework of Section 8, average over a variance that may carry the biology of interest. The healthy human hamstring reference atlas [232] and the equine ageing atlas [207] now make it possible to design experiments that fractionate before measuring, and this should become standard practice.

The single-cell literature also carries its own limitations, which the field has candidly acknowledged: dissociation bias against matrix-embedded cells, low cell yields from dense tissue, small donor numbers and confounding by anatomical site are pervasive [230,231,232]. We return to the site-confound problem in Section 10, because it motivated the computational companion to this review.

**Table 6:** Single-cell and spatial transcriptomic studies of tendon and enthesis, 2020–2026.

Study	Species/Tissue	Method	Principal Finding Relevant to Mechanobiology	Ref.
Kendal et al. 2020	Human (healthy and diseased)	CITE-seq	ITGA7^+^ smooth-muscle-mesenchymal and TPPP3/PRG4^+^ populations; surface markers for isolation	[230]
De Micheli et al. 2020	Mouse Achilles	scRNA-seq	Eleven cell types; pericytes as candidate fibroblast progenitors	[229]
Still et al. 2021	Human progenitor cells under stretch	scRNA-seq	Diseased progenitors show distinct mechanical response with altered mitochondrial biogenesis	[235]
Akbar et al. 2021	Human tendinopathy	scRNA-seq + spatial	Dysregulated immune homeostasis in diseased tendon	[234]
Ackerman et al. 2022	Mouse flexor tendon healing	Lineage tracing + spatial	Molecular programmes of aligned bridging tissue versus peritendinous scar	[240]
Fang et al. 2022	Mouse enthesis	scRNA-seq + lineage tracing	Gli1^+^ mineralising progenitors build the enthesis	[61]
Zhang et al. 2023	Mouse rotator-cuff enthesis	scRNA-seq + spatial	Trajectories of fibrocartilaginous differentiation	[60]
Fu et al. 2023	Human tendinopathy (34,736 cells)	scRNA-seq + spatial	Ten tenocyte subpopulations; inflammation-chondrogenesis-ossification	[231]
Mimpen et al. 2024	Healthy human hamstring	snRNA-seq + spatial	Twelve cell types; fibroblasts as central ECM regulators; reference dataset	[232]
Zamboulis et al. 2024	Equine energy-storing tendon, young vs. old	scRNA-seq	Nine IFM clusters vs. one fascicular; IFM cells preferentially affected by ageing	[207]
Cohen et al. 2026	Human Achilles, enthesis to muscle	Spatially resolved scRNA-seq	Fibroblast specialisation across microanatomy	[233]

ECM, extracellular matrix; IFM, interfascicular matrix; scRNA-seq, single-cell RNA sequencing; snRNA-seq, single-nucleus RNA sequencing.

### 6.2. The Sensor Inventory and Its Evidential Status

**Figure 7:**
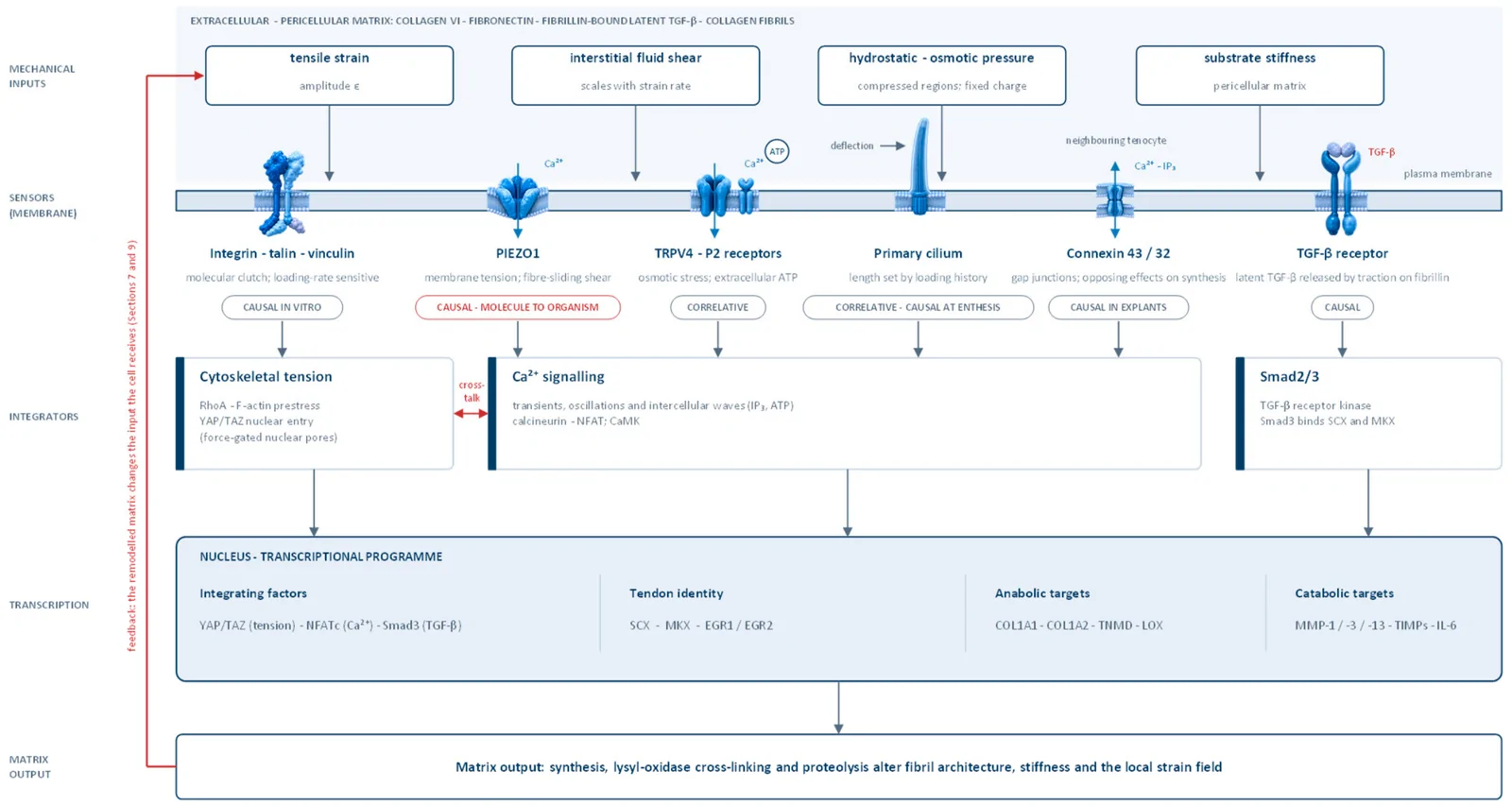
Mechanotransduction in the tenocyte as a layered signal flow. Mechanical inputs (tensile strain, interstitial fluid shear, hydrostatic and osmotic pressure, substrate stiffness) act on six membrane-associated sensor systems; the label beneath each sensor grades the evidence for its role in the tendon (see Table 7). Sensors converge on two integrators-cytoskeletal tension (RhoA, F-actin, YAP/TAZ) and Ca^2+^ signalling (calcineurin–NFAT) and on Smad2/3 downstream of mechanically released TGF-β. These regulate the transcriptional programme (SCX, MKX, EGR1) and its anabolic and catabolic targets. The matrix output alters fibril architecture and stiffness and thereby the input that the cell receives (red loop), closing the cycle discussed in Section 7 and Section 9.

The tenocyte possesses at least six mechanically sensitive input systems (Figure 7): integrin–focal adhesion complexes with force-activated talin and vinculin [36,200,241,242]; stretch-activated PIEZO channels [38,39,243,244]; TRPV4 and related channels responsive to osmotic and mechanical stimuli [245]; the primary cilium [37,246,247,248]; connexin gap junctions and hemichannels [90,239,249]; and nuclear pore complexes deformed by cytoskeletal force [93,250]. The evidence supporting each differs substantially in kind and in strength, and Table 7 grades it.

PIEZO1 is the sensor for which the causal chain from molecule to organism is most complete. Passini and colleagues demonstrated that tenocytes detect the shear generated by collagen-fibre sliding through PIEZO1, that tenocyte-targeted loss or gain of function tunes tendon stiffness in mice, and that humans carrying the E756del gain-of-function variant show increased jumping performance [39]. Nakamichi and colleagues independently showed, using R2482H knock-in mice, that PIEZO1 gain of function produces more compliant, energy-storing tendons and enhanced physical performance [244]. Götschi and colleagues then confirmed the human phenotype directly: E756del carriers had approximately 46% higher patellar tendon stiffness with unchanged tendon dimensions [251]. The two rodent studies differ in the direction of the stiffness change, which has not been resolved; differences in the variants (E756del versus R2482H), in the tendon type examined and in loading history are candidate explanations. In our assessment, the PIEZO1 chain is the single most important advance in tendon mechanobiology of the past five years, because it establishes that a defined channel protein with known gating physics exerts a measurable influence on tissue-level material properties and on whole-organism performance [252].

Integrin adhesions are the best-characterised force-transmission pathway in cell biology generally, and the molecular clutch model in which loading rate, adhesion density and substrate stiffness jointly determine whether force is transmitted [36] makes a specific prediction for tendons: Cells should be sensitive to strain rate independently of amplitude. The prediction is supported. Both the amplitude and frequency of cyclic strain modulate MMP-1 suppression in tendon cells [58], and finite element analysis indicates that strain rate governs fluid-induced shear at the cell surface while amplitude governs cell deformation, so the two channels are physically separable [109]. Disruption of actin abolishes load-dependent MMP-1 regulation [236], and recent work shows that stress deprivation or inhibition of tropomyosin 3.1 reduces F-actin and drives a tendinosis-like phenotype [253], placing cytoskeletal tension upstream of the catabolic programme. The limitation of this literature is that it is almost entirely correlative in vivo. No tenocyte-specific manipulation of integrin or talin function has been performed in loaded tendons.

The primary cilium is an established mechanosensor whose length is set by recent loading history. Stress deprivation causes elongation and cyclic loading prevents or reverses it [246]. Cilia are deflected by tensile load in situ [247], and zonal ciliary elongation correlates spatially with local degradation in stress-deprived tendon [248]. Ciliary length also determines sensitivity, so this constitutes an adaptive gain control implemented in organelle geometry. At the enthesis, the cilium is the nexus at which biophysical stimuli and Hedgehog signalling converge [59]. Its proximal transduction chemistry in tendons remains undefined, and the literature relies on chondrocyte and osteocyte analogies [254,255,256]. No tenocyte-specific primary study of ciliary signalling has appeared since 2022.

Gap junctions give the tenocyte network a distributed regulatory architecture with opposing channels. Blocking connexin 32 reduces load-induced collagen synthesis whereas blocking connexin 43 increases it [239]. Tensile loading suppresses junctional permeability with amplitude-dependent kinetics [92,257]. Calcium waves propagate through this network by inositol trisphosphate diffusion and by extracellular ATP acting on P2 receptors [90,258,259]. This system has received little new primary investigation since 2017, which is a notable gap given its potential to explain how a local mechanical perturbation is converted into a tissue-scale response.

YAP/TAZ and the nucleus integrate cytoskeletal tension into a transcriptional output [40,260,261,262], and force applied to the nucleus opens nuclear pores to accelerate YAP import directly [93]. Computational models of YAP/TAZ mechanotransduction have matured to the point where they can be coupled to tissue-scale mechanics [263], and recent reviews position YAP/TAZ, together with mTOR under overload and GSK3/β-catenin and ERK1/2 under underload as the principal loading-responsive pathways in tendons [264]. Direct tenocyte-specific evidence remains less extensive than that for PIEZO1.

TGF-β occupies a special position because it is stored in the matrix in a latent, mechanically releasable form bound to fibrillin microfibrils [194,265]. Cyclic strain modulates metalloproteinase and matrix gene expression in human tenocytes through TGF-β activation [266]. TGF-β signalling is required for tendon progenitor recruitment in development [267,268] and for tenocyte recruitment during neonatal regeneration [269]. Smad3 integrates TGF-β input with both scleraxis and Mohawk [270]. The matrix thus functions as a pre-loaded chemical signal discharged by mechanics, a route that bypasses transcription entirely in its first step.

**Table 7:** Mechanosensory systems of the tenocyte and the evidential status of each.

Sensor	Stimulus	Strongest Evidence in Tendon	Evidence Tier	Principal Gap	Refs.
PIEZO1	Membrane tension; fibre-sliding shear	Tenocyte-specific gain/loss of function alters tendon stiffness; human variant alters performance and stiffness	Causal, molecule to organism	Direction of stiffness change differs between rodent models	[39,244,251,252]
Integrin–talin–vinculin	Matrix strain; loading rate	Cytoskeletal disruption abolishes load-dependent MMP-1 regulation; rate sensitivity	Causal in vitro; correlative in vivo	No tenocyte-specific adhesion manipulation in loaded tendon	[36,58,109,236,253]
Primary cilium	Bending; deflection	Length responds to loading history; deflection under load; local degradation correlates with elongation	Correlative; causal at enthesis	Transduction chemistry undefined in tendon	[37,59,246,247,248]
Connexin 43/32	Intercellular Ca^2+^ and IP_3_ flux	Opposing effects on load-induced synthesis; permeability regulated by load	Causal in explants	No new primary data since 2017	[90,92,239,257]
TRPV4/P2 receptors	Osmotic stress; extracellular ATP	ATP modulates load-induced catabolic genes; ecto-ATPase induced by load	Correlative	Contribution relative to PIEZO1 unknown	[245,258,259]
YAP/TAZ; nuclear pores	Cytoskeletal tension	Force gates YAP import; models couple to tissue mechanics	Causal in non-tendon cells	Tenocyte-specific in vivo evidence limited	[40,93,263,264]
Latent TGF-β	Cellular traction on fibrillin	Cyclic strain acts via TGF-β activation; required for regeneration	Causal	Kinetics of release under physiological load unmeasured	[194,265,266,269]

### 6.3. Convergence and the Question of Rate Limitation

Several sensors converge on cytosolic Ca^2+^, which encodes information in amplitude, frequency, spatial localisation and wave propagation [90,255,258], and calcineurin–NFAT, which signals downstream of Ca^2+^ cooperates with scleraxis at the *COL1A1* promoter [271]. Others converge on RhoA-dependent cytoskeletal tension and YAP/TAZ. There are therefore at least two parallel routes from matrix strain to gene expression: a biochemical cascade operating over seconds to minutes, and a direct mechanical route-matrix, integrin, cytoskeleton, nuclear envelope, nuclear pore-operating at the speed of force transmission [93,250].

The question that this convergence raises and that the literature has not answered is which input is rate-limiting for the transcriptional response under physiological loading. Redundancy is one possibility, and would be biologically sensible for a tissue whose homeostasis depends on load detection. Division of labour by stimulus modality is another: PIEZO1 responds to fibre-sliding shear [39], adhesions to strain rate [36,109], the cilium to sustained deflection [247], and TGF-β release to traction against the pericellular matrix [194]. If the latter is correct, then the “mechanical stimulus” of Section 5.4 is not one variable but several, each read by a different sensor, and the non-monotonic dose–response of Section 9 may arise because different sensors dominate in different loading regimes. This is a testable hypothesis, and the tools to test it tenocyte-specific channel knockouts [39,244], explant systems with controlled loading [226,227] and single-cell readouts [232,235] now exist.

### 6.4. Transcriptional Identity

The transcriptional programme that defines the tenocyte is comparatively well established. Scleraxis is the earliest and most specific marker; it is required for the differentiation of force-transmitting tendons while muscle-anchoring tendons are comparatively spared, indicating that the programme is itself specialised by mechanical function [272,273,274]. Scleraxis directly activates tenomodulin [275,276] and cooperates with NFATc on *COL1A1* [271]. Mohawk-null mice have hypoplastic tendons with small fibrils [277,278,279]. EGR1 and EGR2, immediate-early genes inducible within minutes of stimulation, direct tendon differentiation and promote repair [94,280,281]. Tenomodulin is required for tenocyte proliferation and tendon maturation [282]. Scleraxis expression is regulated by TGF-β and by mechanical load [267,268], closing a loop in which load activates TGF-β, which drives scleraxis, which drives the matrix genes that build the structure transmitting the load.

Lineage tracing has complicated the picture in an informative way. Depletion of scleraxis-lineage cells in adult mouse tendons paradoxically improved healing while disrupting homeostasis, indicating that this lineage is required for matrix maintenance but contributes to the fibrotic component of repair [283]. Scleraxis-lineage cells form the organised bridging tissue during healing [284], and Tppp3^+^Pdgfra^+^ progenitors contribute to both regeneration and fibrosis through a shared PDGF signalling node [285]. The transcriptional identity of the tenocyte is thus not a fixed state but a position in a landscape whose trajectories are set jointly by mechanical and biochemical inputs.

### 6.5. Critical Appraisal of Transition IV

The stimulus-to-transcription transition has advanced more than any other since 2021, principally through PIEZO1 and through single-cell atlases. It remains, however, the transition with the largest number of parallel candidate mechanisms and the least quantitative coupling between them. Three deficiencies are decisive for the modelling programme. First, the relationship between any mechanical input variable and any transcriptional output has not been measured with the precision a quantitative model requires. There is no published transfer function from cyclic strain amplitude to a Ca^2+^ transient frequency or from fluid shear to YAP nuclear fraction in tendon cells in situ. Second, the relative contributions of the sensors under physiological loading are unknown. Third, the cellular heterogeneity revealed by single-cell methods has not yet been incorporated into any mechanobiological experiment or model. The last point is the most tractable and, we suggest, the most consequential.

## 7. Transition V: From Cellular Output Back to the Matrix

### 7.1. The Cell Templates the Structure It Will Inhabit

The classical presentation of a structural hierarchy is bottom-up. It is incomplete for tendons because the cell controls the assembly of every level above the molecule. Procollagen is synthesised with hydroxylation and glycosylation completed on unfolded chains before triple-helix formation, so that folding rate and modification extent compete kinetically. This competition is a control point for collagen quality [49,111,130,286]. Propeptide cleavage by ADAMTS2 and BMP1 renders the molecule self-assembling only after secretion, at a location the cell determines [49,112]. Birk and Trelstad described extracellular compartments formed by the plasma membrane within which fibril, bundle and macroaggregate formation occur under cellular control [51]. Canty, Kadler and colleagues identified fibripositors, tension-dependent membrane protrusions containing single fibrils whose co-alignment specifies the parallel architecture of the tissue [52,287]. Postnatal growth proceeds by a structure-based expansion mechanism in which existing fibrils lengthen within a maintained architecture [176,288]. Crimp itself is generated by tenocyte contraction [205]. Cross-link installation by lysyl oxidase is mechanoresponsive [39,54]. The cell therefore sets fibril orientation, fibril length distribution, crimp and cross-link density, precisely the structural parameters that determine the strain field it will subsequently experience.

### 7.2. Turnover: A Matrix Built Once

The rate at which this control is exercised in adult tissue is very low. Radiocarbon bomb-pulse dating shows that the collagen core of a healthy adult human Achilles tendon is essentially not renewed after skeletal maturity [97], and aspartic acid racemisation confirms the accumulation of long-lived collagen with age [144]. The same method applied to tendinopathic tissue produced an unexpected and important result: Approximately half of the collagen in a tendinopathic tendon had undergone slow renewal over the years preceding symptoms, implying that abnormally high turnover precedes clinical disease rather than following it [98]. Microdialysis and stable-isotope studies nonetheless detect measurable collagen synthesis after exercise in peritendinous tissue [11,289], and postnatal loading drives adaptation primarily through the non-collagenous matrix [198]. The reconciliation is that turnover is concentrated in the interfascicular and peritendinous compartments and in the non-collagenous matrix, while the fascicular collagen core is nearly static [4,10,198]. Tendons thus operate on an unusual economy build once, maintain minimally that is adaptive for a tissue operating near the oxygen diffusion limit [69,129,225] but that leaves the core matrix exposed to cumulative, irreversible chemical decay (Section 3.5).

### 7.3. Proteolysis and Its Mechanical Modulation

Matrix metalloproteinases and ADAMTS proteases, controlled by transcription, zymogen activation and tissue inhibitors, execute degradation [56,290,291]. Altered MMP activity accompanies tendon pathology [55,292,293,294]; the homeostatic relevance of the system is demonstrated by explant studies. Stress deprivation upregulates MMP-1 and produces measurable degradation, whereas modest cyclic strain suppresses it [58,236] and upregulates collagen synthesis [237,238].

The finding that most changes the interpretation of this system is that mechanical strain modulates enzymatic cleavage at the molecular level, independently of any cellular response. Collagenase must locally unwind the triple helix before hydrolysing the peptide bond [295]. Axial tension opposes unwinding, and low forces slow cleavage of the collagen monomer [296] while strain stabilises reconstituted fibrils against MMP-8 [297] and fibrillar architecture governs accessibility of the cleavage site [161]. A single-molecule study reporting the opposite load accelerating MMP-1 cleavage by two orders of magnitude [298] is most plausibly reconciled by the distinction between an isolated monomer, in which force can favour partial unfolding, and a molecule embedded in a cross-linked fibril, in which packing shields the site and tension resists unwinding. The principle that emerges is that the matrix is a mechanically tuned substrate for its own degradation: loaded collagen is protected and unloaded collagen is vulnerable. This provides a physicochemical, cell-independent mechanism for use-dependent maintenance that operates in parallel with, and reinforces, the cellular pathways of Section 6, and it offers a molecular explanation for stress-deprivation degeneration [58,227,236] and for preferential degeneration of locally under-loaded regions [248].

### 7.4. Ageing, Senescence and the Loss of Remodelling Competence

Ageing alters the output side of the loop as much as the matrix. The transcriptome of an uninjured aged human Achilles tendon differs systematically from young tissue [299]. An aged tendon responds to load maladaptively through an active MMP-dependent process rather than accumulating passive damage alone [300], and aged tendons exhibit altered mechanisms of strain-dependent matrix remodelling [301]. Senescence is now implicated directly: induced senescence in tendon explants shifts remodelling toward degradation under unloading [302], and senescent and inflammatory signatures are concentrated in the interfascicular cell populations of an aged energy-storing tendon [207], which identifies the compartment in which senolytic or senomorphic intervention would have to act. Moderate exercise mitigates age-related deterioration of tendon stem cells [303], indicating that corrective capacity is retained if the stimulus is placed appropriately.

Metabolically, the tenocyte is glycolytic and adapted to low oxygen [69,129,225,304]. Hypoxia regulates early human tendinopathy [129] and reactive oxygen species act both as damaging agents and as signals [305,306,307]. The plausible integration is that overload increases metabolic demand and oxidative stress in a tissue with limited antioxidant reserve and minimal capacity to replace damaged matrix, and that senescence of the responding cells converts a once-corrective response into a degradative one.

### 7.5. Critical Appraisal of Transition V

The output transition is well characterised qualitatively and almost entirely unquantified. Rate constants for synthesis, cross-link installation and proteolysis as functions of the cellular state are not available for tendons; the Bell-type strain dependence of proteolysis (Section 8.7) has been demonstrated at the molecular level but not parameterised in tissue; and the coupling between senescence and matrix output is described in explants but not in vivo. The single most useful measurement for closing the loop would be a simultaneous determination, in a loaded explant system, of local matrix strain, the transcriptional state of identified cell subpopulations and the rates of collagen synthesis and degradation over days to weeks. Such an experiment is feasible with current explant platforms [226,227] and stable-isotope labelling, and it would supply the parameters that every remodelling model currently borrows or assumes.

## 8. A Coupled Mathematical Framework 

### 8.1. Requirements

**Figure 8:**
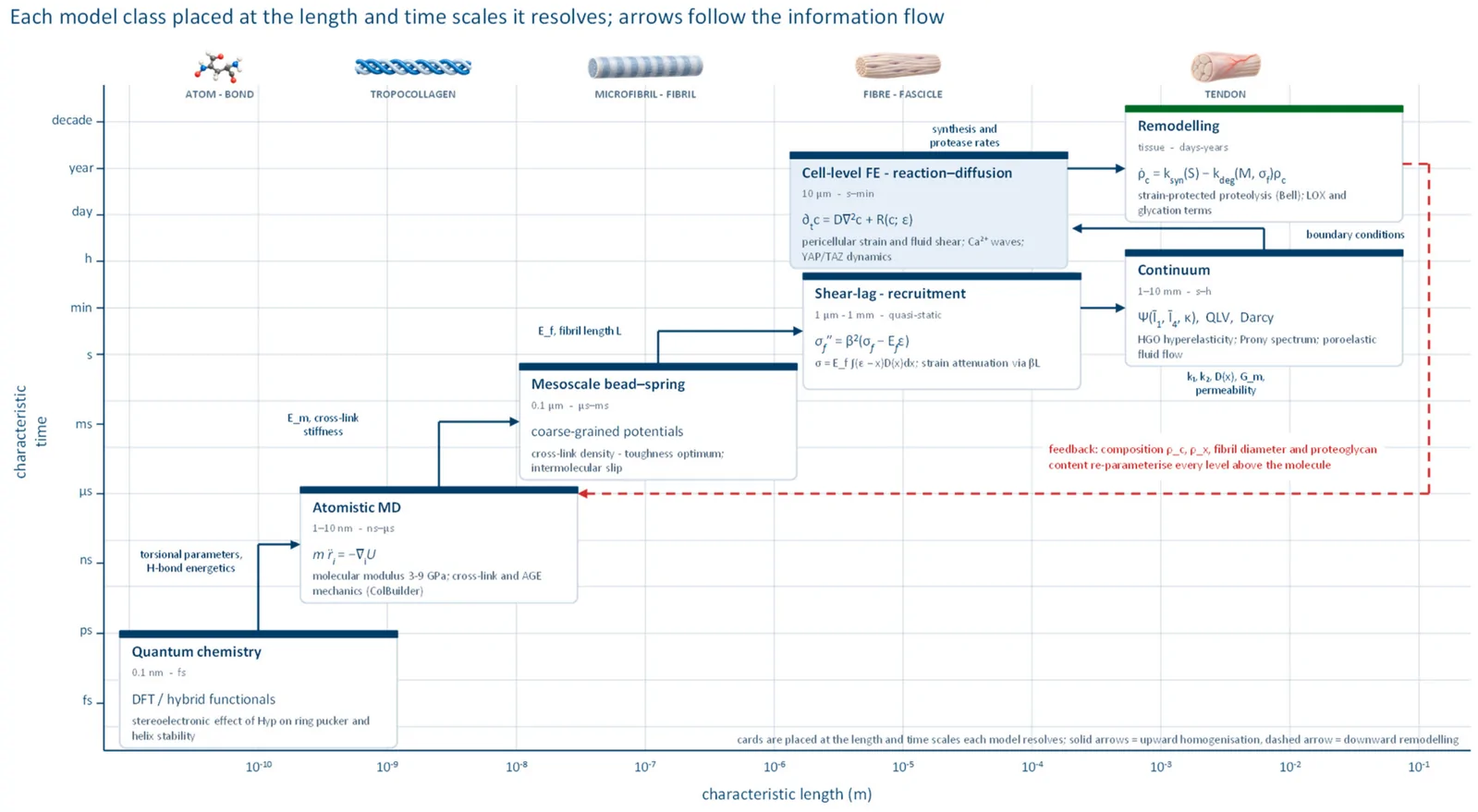
Multiscale modelling architecture for the tenocyte–matrix unit shown as a scale map. Each model class is placed at the characteristic length (abscissa, logarithmic) and time (ordinate, logarithmic) that it resolves, together with its governing relation. Solid arrows follow the upward flow of information by homogenisation and are labelled with the parameters passed between levels (torsional and hydrogen-bond energetics; molecular modulus and cross-link stiffness; fibril modulus and length; recruitment density, matrix shear modulus and permeability; cell-level boundary conditions; synthesis and protease rates). The dashed arrow is the downward coupling, almost universally absent from published models, by which cell-mediated remodelling re-parameterises every level above the molecule. DFT, density functional theory; FE, finite element; HGO, Holzapfel–Gasser–Ogden; MD, molecular dynamics; QLV, quasi-linear viscoelasticity.

A mathematical description adequate to the five transitions must represent deformation at each level with experimentally measurable variables; pass information between levels through physically justified coupling relations rather than fitted correction factors; represent time dependence from milliseconds to years; accommodate the feedback from cellular output to matrix composition; and reduce, in appropriate limits, to the single-scale models that already fit data. No published model meets all five requirements. What follows is an inventory of the validated components, the equations that define them and the interfaces between them (Figure 8, Table 8). We include equations because a verbal claim that “cross-links stiffen the fibril” is compatible with almost any data, whereas a model with an explicit cross-link density parameter that predicts a specific modulus increment is not [104].

### 8.2. Continuum Kinematics and Anisotropic Hyperelasticity

At the tissue level, the deformation gradient F defines the right Cauchy–Green tensor C = F^T^F with invariants I_1_ = tr C and, for a fibre direction a_0_, the pseudo-invariant I_4_ = a_0_·C a_0_ = λ_f_^2^, the squared fibre stretch. Tendon is a one-fibre-family material and I_4_ is the dominant kinematic variable. The Holzapfel–Gasser–Ogden strain energy function, developed for arterial walls and now standard for tendons and ligaments, is [107,308]

$$\Psi =\frac{\mu }{2}\left({\bar{I}}_{1}-3\right)+\frac{k_{1}}{2k_{2}}\left\{\exp\left[k_{2}{\left\langle {\bar{I}}_{4}-1\right\rangle }^{2}\right]-1\right\}+U\left(J\right)$$

in which the first term is the isotropic ground substance with shear modulus μ, the second the exponentially stiffening collagen fibres with stress-like parameter k_1_ and dimensionless k_2_. The Macaulay brackets enforce tension-only fibre loading. U(J) is the volumetric term. The dispersion extension replaces I_4_ by κ I_1_ + (1 − 3κ)I_4_, with κ = 0 for perfectly aligned and κ = 1/3 for isotropic fibres [308]. Because tendinopathic collagen is disorganised, κ is a natural pathological parameter. The Cauchy stress follows as σ = (2/J) F(∂Ψ/∂C)F^T^, and finite element implementations for transversely isotropic incompressible tissues are mature [309,310]. Shearer derived a strain energy function from fascicle microstructure rather than by fitting, giving parameters with structural meaning, and later showed that the helical fibril arrangement within fascicles accounts for the relative compliance of energy-storing tendons [106,311]. This is the direction of travel we advocate: parameters that are structural measurements rather than fitting constants, because only such parameters can be updated by a remodelling model.

### 8.3. Fibril Recruitment

The toe region arises from progressive recruitment of crimped fibres. Let D(x) dx be the fraction of fibres that become taut between strain x and x + dx. A fibre recruited at strain x carries strain ε − x and stress E_f_(ε − x), so the tissue stress is [2,312,313]

$$\sigma \left(\varepsilon \right)=E_{f}\int_{0}^{\varepsilon }\left(\varepsilon -x\right)\;D\left(x\right)\;\mathrm{dx},\;\;\frac{d^{2}\sigma }{d\varepsilon^{2}}=E_{f}D\left(\varepsilon \right)$$

The second derivative of the measured stress–strain curve is proportional to the recruitment density, which converts a mechanical measurement into a structural one that can be tested against optical measurements of crimp recruitment [203]. Once all fibres are recruited, the tangent modulus saturates at E_f_ and the response becomes linear without further assumption. Recruitment formalisms have been extended to include progressive fibre damage [314].

### 8.4. Quasi-Linear Viscoelasticity and Its Limits

Fung’s quasi-linear theory assumes that the relaxation function factorises into a reduced relaxation function G(t), with G(0) = 1, and an instantaneous elastic response σ^e^(ε) [41,42]:

$$\sigma \left(t\right)=\int_{-\infty }^{t}G\left(t-s\right)\;\frac{\partial \sigma^{e}\left[\varepsilon \left(s\right)\right]}{\partial \varepsilon }\;\frac{\mathrm{d\varepsilon}}{\mathrm{ds}}\;\mathrm{ds},\;\;G\left(t\right)=g_{\infty }+\sum_{i=1}^{N}g_{i}\exp\left(-t/\tau_{i}\right)$$

For tendon, three Prony terms with τ_1_ ≈ 5–10 s, τ_2_ ≈ 60 s and τ_3_ ≈ 500–1000 s typically suffice (Figure 5B), and these can be tentatively mapped onto interfibrillar shear and fast fluid redistribution, poroelastic flow and fibril sliding, and slow water redistribution, respectively [30,80,91]. The separability assumption fails when relaxation rate depends on strain magnitude, as observed at higher strains. Nonlinear formulations with strain-dependent spectra describe this better [315,316]. Anisotropic finite element implementations exist [220]. Constitutive artificial neural networks that discover the functional form of the strain energy from data while preserving thermodynamic constraints have recently been demonstrated for soft fibrous tissue [317,318]. They are the most promising route to tendon models that are simultaneously flexible and physically admissible, provided the discovered forms are constrained to structurally interpretable terms.

### 8.5. Shear-Lag Load Transfer

If fibrils are finite (Section 4.3), load enters each through interfacial shear. Force balance on a fibril of radius r and length L embedded in a matrix of shear modulus G_m_ and effective thickness h gives

$$\frac{d^{2}\sigma_{f}}{dz^{2}}=\beta^{2}\left(\sigma_{f}-E_{f}\varepsilon \right),\;\;\sigma_{f}\left(z\right)=E_{f}\varepsilon \left[1-\frac{\cosh\left(\mathrm{\beta z}\right)}{\cosh\left(\mathrm{\beta L}/2\right)}\right],\;\;\beta =\sqrt{\frac{2G_{m}}{r\;h\;E_{f}}}$$

The dimensionless group βL controls transfer efficiency. For βL ≫ 1, the fibril reaches the affine stress E_f_ε over most of its length, whereas for βL ≲ 1 it is never fully loaded and deformation is accommodated by interfibrillar shear. This single group quantifies the strain attenuation of Figure 5A in terms of measurable structural quantities [31,32,170], and it settled the glycosaminoglycan controversy by establishing what parameter values would be required for that hypothesis to hold [186]. Its interpretation depends on the fibril continuity question: if fibrils are millimetres long, βL is large at short gauge lengths and the model reduces to affine loading, consistent with the gauge length dependence observed experimentally [34].

### 8.6. Poroelasticity and the Cell-Level Environment

A tendon is a fluid-saturated porous solid. With Darcy flux w = −k∇p, total stress σ = −pI + σ^s^_eff_ and the effective solid stress from Section 8.2, the biphasic formulation yields a pressure diffusion equation with characteristic time τ_p_ ~ L^2^/(H_A_ k), where H_A_ is the aggregate modulus and L the drainage length [81,82,219]. Low permeability and long drainage paths give poroelastic times of minutes, consistent with the intermediate Prony term. This formulation is what converts tissue loading into cell-level stimuli: the finite element model of Lavagnino and colleagues predicted that strain rate governs fluid shear at the cell surface while amplitude governs cell deformation [109], following the cell-in-matrix methodology established for chondrocytes [110,319]. The tendon literature lacks models that incorporate measured pericellular matrix properties and fibril-scale heterogeneity; the local strain amplification demonstrated in fibrocartilage [223] indicates that this omission may be quantitatively serious.

### 8.7. Signalling and Turnover

Intercellular calcium waves and paracrine signalling are reaction–diffusion systems [320,321]

$$\frac{\partial c_{i}}{\partial t}=D_{i}\nabla^{2}c_{i}+R_{i}\left(c_{1},\ldots ,c_{n};\varepsilon ,\dot{\varepsilon }\right),\;\;J_{\mathrm{mech}}=J_{\max}\frac{{\left(\varepsilon /\varepsilon_{50}\right)}^{n}}{1+{\left(\varepsilon /\varepsilon_{50}\right)}^{n}}$$

in which the mechanical input enters the reaction terms through Hill-type activation of mechanosensitive channels with half-activation strain ε_50_ and cooperativity n, and gap-junction coupling enters as a flux boundary condition whose permeability is itself load-dependent [92,257]. Computational models of YAP/TAZ signalling of this general class have been reviewed recently [263] and are ready to be coupled to the tissue-level formulations above.

Matrix composition evolves on the slowest time scale. A minimal formulation tracks collagen density ρ_c_ and cross-link density ρ_x_:

$$\frac{d\rho_{c}}{\mathrm{dt}}=k_{\mathrm{syn}}\left(S\right)-k_{\deg}\left(M,\sigma_{f}\right)\;\rho_{c},\;\;k_{\deg}=k_{0}\;M\exp\left(-\frac{\sigma_{f}\Delta V^{‡}}{k_{B}T}\right)$$

where S is an integrated mechanical stimulus, M is the active protease concentration, and the exponential factor introduces a Bell-type dependence of degradation on fibril stress σ_f_ through an activation volume ΔV^‡^ that represents the local unwinding required for cleavage [295,296,297]. An analogous equation for ρ_x_ carries a lysyl-oxidase term dependent on S and a glycation term proportional to glucose concentration. Multiscale remodelling models of this kind have been constructed for tendon healing, coupling musculoskeletal loading to collagen deposition [46], and the critical structural feature that k_syn_, k_LOX_ and M are functions of the cellular state, which depends on the local mechanics, which in turn depend on ρ_c_ and ρ_x_ closes the loop. The stability properties of this loop, rather than the properties of any individual term, determine whether the tissue is homeostatic, hypertrophic or degenerative.

**Table 8:** Mathematical models applicable to the tenocyte–matrix system, with their validated domains and principal limitations.

Model Class	Governing Relation	Scale/Time Scale	Strength	Limitation	Refs.
Quantum chemistry	Electronic structure	<1 nm; fs	Explains stereoelectronic effects	Cannot reach mechanical scales	[101,124]
All-atom molecular dynamics	Newton’s equations with force field	1–100 nm; ns–µs	Derives molecular mechanics from chemistry; AGE cross-links now insertable	Time-scale limited; force-field dependent	[7,43,44,103,147]
Coarse-grained/mesoscale	Bead–spring with effective potentials	0.1–1 µm; µs–ms	Cross-link density–toughness relation	Loses chemical detail	[7,104]
Worm-like chain	Interpolation formula for F(x)	Single molecule	Entropic elasticity	Low force only	[102,153]
Shear-lag	σ_f_″ = β^2^(σ_f_ − E_f_ε)	Fibril–fascicle; quasi-static	Strain attenuation from structural parameters	Requires finite fibrils; one-dimensional	[31,32,34,186]
Fibril recruitment	σ = E_f_∫(ε − x)D(x)dx	Fascicle; quasi-static	Structurally interpretable toe region	No time dependence alone	[2,312,313,314]
Quasi-linear viscoelasticity	Convolution with G(t)	Tissue; s–h	Standard; fits relaxation and creep	Separability fails at high strain	[41,42,220]
Nonlinear viscoelasticity	Strain-dependent spectrum	Tissue	Captures strain-dependent relaxation	Identification harder	[315,316]
Anisotropic hyperelasticity (HGO)	Ψ(I_1_, I_4_, κ)	Tissue; quasi-static	Three-dimensional, FE-ready, dispersion parameter	Phenomenological parameters	[107,308,309]
Microstructural hyperelasticity	Ψ from fascicle geometry	Fascicle–tissue	Structural parameters	Requires morphometry	[106,311,322,323]
Constitutive neural networks	Thermodynamically constrained learned Ψ	Tissue	Automated model discovery	Interpretability must be enforced	[317,318]
Biphasic/poroelastic	Darcy flow + momentum balance	Tissue and cell; s–min	Predicts fluid shear on cells	Permeability rarely measured	[81,82,219]
Cell-in-matrix FE	Poroelastic FE with explicit cell	Cell; s–min	Converts tissue load to cell stimuli	Pericellular properties unknown	[109,110,319]
Reaction–diffusion	∂_t_ c = D∇^2^ c + R(c; ε)	Cell network; s–min	Intercellular waves; YAP/TAZ dynamics	Parameters from other tissues	[90,263,320,321]
Remodelling ODE/multiscale healing	$\dot{\rho }$ = k_syn_(S) − k_deg_(M, σ_f_)ρ	Tissue; days–years	Closes the loop	Rate constants unconstrained	[45,46]

FE, finite element; HGO, Holzapfel–Gasser–Ogden.

### 8.8. Coupling Strategy and a Caution

The upward path in Figure 8 is parameter passing by homogenisation. Atomistic simulation supplies cross-link stiffness and molecular modulus to mesoscale fibril models, which supply E_f_ to shear-lag and recruitment models, which in turn supply k_1_, k_2_ and D(x) to the continuum strain energy function. The continuum model then supplies boundary conditions to the cell-level poroelastic model. The downward path is that cellular output modifies ρ_c_, ρ_x_, fibril diameter distribution and proteoglycan content, thereby altering the parameters at every higher level. Implementing the downward path requires only that the turnover equations be coupled to the parameter definitions of the structural models, but it requires those parameters to be structural rather than phenomenological, which is why the microstructurally derived strain energy functions matter [106,311,322,323].

A model with fifty parameters, thirty of them unmeasured, is a curve-fitting exercise rather than a theory. The value of the framework lies in the specific and falsifiable claim that a small set of measurable structural quantities cross-link density, fibril diameter distribution and length, crimp recruitment density, interfibrillar and interfascicular shear moduli, permeability and pericellular stiffness suffices to determine tissue mechanics and the cellular mechanical environment.

## 9. Synthesis: The Tenocyte as Controller of a Closed Loop

### 9.1. The Causal Chain Restated

The five transitions can now be connected into a single sequence, each link of which is supported by the evidence reviewed above. Prolyl 4-hydroxylase installs a hydroxyl whose stereoelectronic effect pre-organises the polyproline-II backbone and raises the triple-helical melting temperature by approximately 30 K, converting a marginally unstable molecule into one that is secreted rather than degraded [24,25,26,122,125]. The cell processes, nucleates and orients the resulting collagen within fibripositors, installs telopeptide cross-links through lysyl oxidase, and thereby fixes the fibril orientation, length distribution and cross-link density that will determine its own mechanical environment [51,52,54,287]. Quarter-staggered packing produces the D-period and a cross-link network whose density sets a stiffness–toughness trade-off with an interior optimum [8,9,104]. Load enters finite fibrils by interfibrillar shear with efficiency governed by βL, crimp removal generates the toe region, and strain is attenuated at every level so that the molecule experiences roughly a tenth of the tissue strain [2,27,29,30,31,34]. The tenocyte reads a filtered local strain, a fluid shear proportional to strain rate, and hydrostatic and osmotic loads through PIEZO1, adhesions, the cilium and connexin channels, which converge on Ca^2+^, cytoskeletal tension, YAP/TAZ and released TGF-β and thence on scleraxis, Mohawk and EGR1 [39,40,93,194,239,246,273]. The transcriptional output collagen, lysyl oxidase, proteoglycans and proteases modifies every structural parameter above it, while strained collagen independently resists proteolysis so that the matrix protects itself in proportion to the load it carries [295,296,297]. The loop is closed at two levels: one cellular and slow, one physicochemical and immediate.

### 9.2. The Tendon Mechanostat

**Figure 9:**
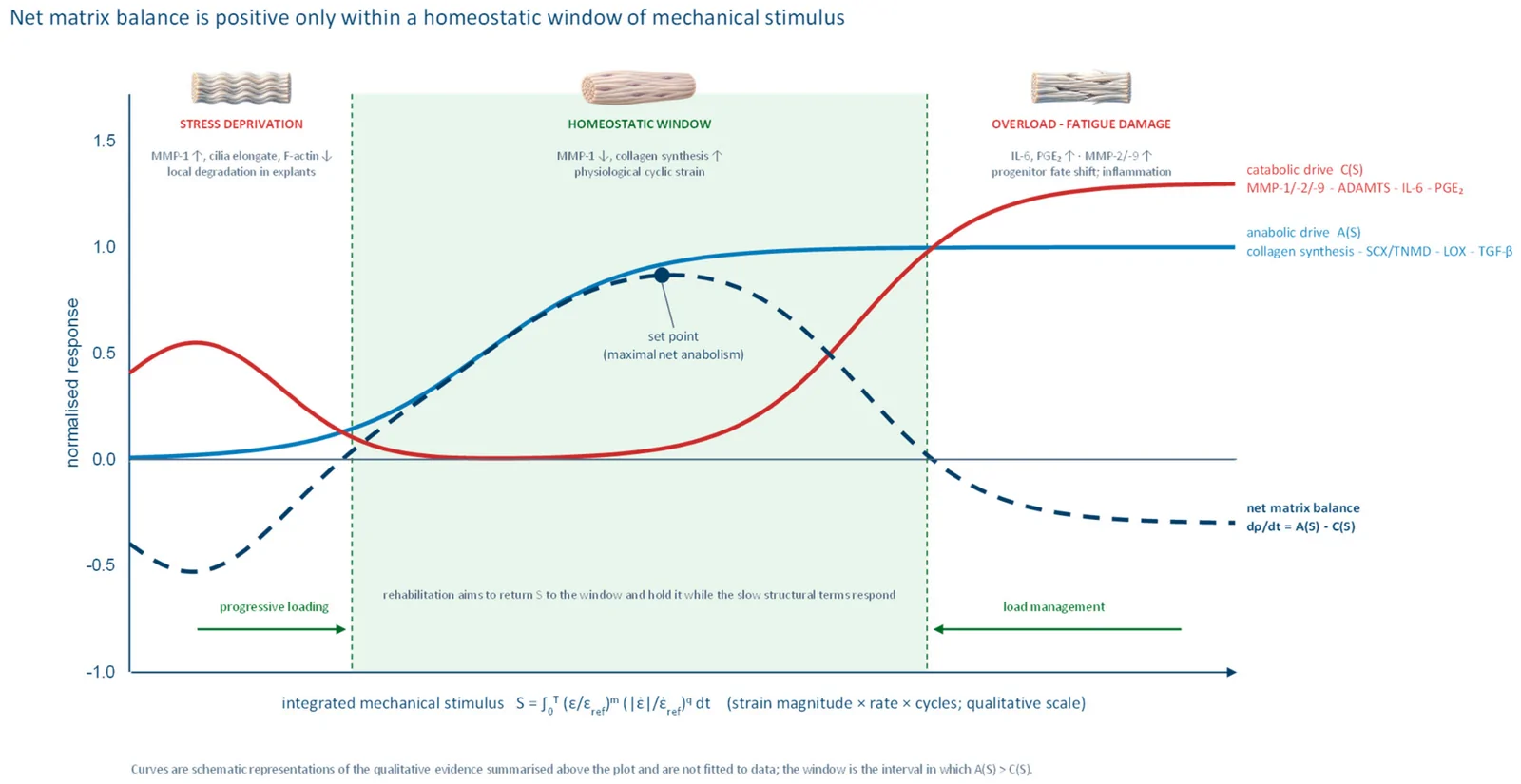
The tendon mechanostat. Anabolic drive A(S) (collagen synthesis, scleraxis and tenomodulin expression, lysyl oxidase, TGF-β signalling) is sigmoidal in the integrated mechanical stimulus S; catabolic drive C(S) (MMP-1/-2/-9, ADAMTS, IL-6, PGE_2_) is elevated under stress deprivation and under overload. Net matrix balance dρ/dt = A(S) − C(S) is positive only within the homeostatic window (green), which contains a set point of maximal net anabolism; the experimental observations supporting each regime are listed above the plot. Rehabilitation strategies (progressive loading, load management) aim to return S to the window. The curves are schematic representations of the qualitative evidence cited in Section 9.2 and are not fitted to data.

The behaviour of this loop is captured by a dose–response relationship that is non-monotonic in mechanical stimulus (Figure 9). We adopt the term mechanostat by analogy with bone, noting that the tendon version differs in having a far slower structural response and a far less renewable matrix. Defining an integrated stimulus S and the net matrix balance as

$$S=\int_{0}^{T}{\left(\frac{\varepsilon \left(t\right)}{\varepsilon_{\mathrm{ref}}}\right)}^{m}{\left(\frac{\left|\dot{\varepsilon }\left(t\right)\right|}{{\dot{\varepsilon }}_{\mathrm{ref}}}\right)}^{q}\;\mathrm{dt},\;\;\frac{\mathrm{d\rho}}{\mathrm{dt}}=A\left(S\right)-C\left(S\right)$$

where the anabolic term A(S) is sigmoidal and saturating and the catabolic term C(S) is U-shaped elevated under stress deprivation and rising again under overload we define the homeostatic window as the interval in which A > C. The evidence for each branch is direct. Under deprivation, explants upregulate MMP-1 and lose mechanical properties [58,236], cilia elongate and local degradation follows [246,248], F-actin loss drives a tendinosis-like phenotype [253], and the response depends on the pathophysiological niche and can be blocked pharmacologically through ERK1/2 [227,324]. Within the window, modest cyclic strain suppresses MMP-1 and raises collagen synthesis [58,237,238]. Under overload, fatigue loading raises IL-6 [325], cell shape and inflammatory and degradative markers change [213], PGE_2_ rises and drives progenitors toward non-tenogenic fates [326], and gelatinases increase in rupture [294].

The question posed by Arnoczky, Lavagnino and Egerbacher whether tendinopathy results from over- or under-stimulation of tendon cells [57] is answered by the mechanostat as both, because the same effector machinery is engaged at both extremes. This resolves the paradox that both rest and excessive loading worsen tendinopathy, and it supplies the theoretical rationale for progressive loading rehabilitation, whose aim is to return S to the window and hold it there while the slow structural terms respond [17,18,19].

### 9.3. Why the Loop Fails in Tendons Specifically

Three features make this loop unusually fragile. First, because the collagen core is essentially not renewed in adults [97,144], damage integrates over decades and a corrective term that acts on a time scale of decades cannot reject a perturbation acting over months. Second, because AGE cross-links accumulate as a function of time and glycaemia and are removed only by turnover, the loop contains an integrating disturbance with no reset [96,139,327]. Third, because strain is attenuated non-uniformly and tendon geometry creates regions of compression and low tension, some regions sit at low S while the organ as a whole is loaded, so that focal degeneration coexists with adequate global loading [100,225,248].

### 9.4. Pathological Consequences

Tendinopathy is characterised by collagen disorganisation, increased type III collagen, proteoglycan accumulation, neovascularisation, altered cellularity and a genuine if atypical inflammatory component [18,19,328,329]. In the framework above, disorganisation corresponds to an increase in the dispersion parameter κ of Section 8.2, which reduces effective fibre stiffness and shifts load onto fewer aligned fibres, a positive feedback toward further damage. The continuum model of Cook and Purdam organises the clinical course into reactive, dysrepair and degenerative stages that differ in reversibility [99,330], and the ^14^C evidence that abnormally high turnover precedes symptoms by years [98] places the point of intervention earlier than clinical presentation. Single-cell studies now describe the disease as a progressive reorganisation of the resident population toward inflammatory, chondrogenic and ossifying states [231,234], and the recent primers on Achilles tendinopathy and rotator cuff disease frame management in terms compatible with the loading-window concept [19,62].

Ageing affects nearly every level: AGE accumulation restricts sliding [96,141,142,146], interfascicular elastin becomes disorganised and interfascicular fatigue life falls more than fascicular fatigue life [73,206], collagen damage accumulates [144], the aged transcriptome is altered [299], remodelling competence is lost through senescence [301,302], and the cells most affected are those of the interfascicular matrix [207]. The convergence of ageing pathology on the interfascicular matrix the compartment that governs energy-storing function explains why it is energy-storing tendons that rupture.

Diabetes accelerates the Maillard chemistry of Section 3.5 directly, and the predicted phenotype of stiffer, less extensible and less tough tendons with suppressed sliding is observed [140,141,142,143,151,327]. The human evidence that collagen disorganisation predicts mechanics better than AGE content [148], and that diabetic dysfunction extends to nanoscale plasticity and RAGE-mediated cellular effects [149,152], means that AGE-directed intervention alone is unlikely to be sufficient.

Healing is fibrotic rather than regenerative [331,332,333]. Lineage tracing and spatial transcriptomics have identified the responsible populations: scleraxis-lineage cells form organised bridging tissue but also contribute to fibrosis [283,284], Tppp3^+^Pdgfra^+^ progenitors share a PDGF node between regeneration and fibrosis [285], TGF-β is required for neonatal regeneration [269], and the spatial–molecular map of healing has been defined [240]. From the loop perspective, healing fails because the plant and the controller are simultaneously abnormal: the repair matrix has different properties, so a given load produces a different local stimulus, and the responding cells have different set points. Restoring load alone cannot restore structure.

## 10. Computational Companion: Auditing What Public Tendon Omics Can Support

A synthesis of this kind generates hypotheses that identified cell subpopulations differ in mechanosensitivity, that specific gene modules track the mechanostat and that mechanistic model variables have transcript correlates which the published tendon transcriptome might be expected to test. To determine whether it can, we built an open-source companion, tenocyte-virtual-cell, released under the MIT licence [334]. The repository is a Python package with a command-line interface that acquires and harmonises public tendon expression datasets, runs batch and confound diagnostics, evaluates predictive models under leave-one-study-out and leave-one-patient-out cross-validation against explicit null baselines, applies permutation testing and bootstrap stability selection, infers gene-regulatory networks with tree-based methods and positive- and negative-control prior edges (for example, scleraxis-tenomodulin, Smad3-scleraxis) and compares the ordinal predictions of a mechanistic tenocyte model against measured transcript changes.

Its principal result is cautionary. On the most reused human tendinopathy dataset (GSE26051; supraspinatus lesion versus subscapularis control from the same patients [335]), a classifier given only the anatomical site of the biopsy, with no expression data, achieved a pooled area under the receiver operating characteristic curve of 0.93, exceeding the best whole-transcriptome model (0.89) and the best mechanistic gene-panel model (0.86). The reason is a within-patient site confound: all lesional samples derive from one tendon and all controls from another, and only a minority of patients contributed both biopsies from the same tendon. A site-only baseline of this kind is, to our knowledge, not reported in the studies that reuse this dataset, and its omission means that classifiers trained on it may learn anatomy rather than disease. The companion also documents that only fourteen of the twenty-five state variables of the mechanistic model have an unambiguous transcript proxy, which bounds how far any transcriptomic dataset can validate a mechanobiological model of this class.

Three limitations must be stated. First, the repository is a development snapshot without a versioned release or archival identifier, and its predictive-model demonstrations are run on synthetic calibration cohorts by design, with a guard that prevents such outputs being presented as biological findings; the site-confound audit is the only result derived from real data. Second, most manifest accessions beyond the bulk datasets remain to be verified against live repositories. Third, the analysis is confined to transcriptomics, whereas the mechanostat operates through post-translational and physicochemical mechanisms that transcripts do not report. We include the companion not as a source of findings but as an explicit, reproducible statement of what the field’s public data can and cannot currently support, in the same spirit as the recent perspectives on obstacles in tendon omics [230,232].

## 11. Conclusions and Future Perspectives

The tenocyte and its matrix constitute a single mechanochemical system whose behaviour can be traced without conceptual discontinuity from the stereoelectronic effect of a hydroxyl group to the failure of an Achilles tendon. Four conclusions follow from the evidence examined here.

First, the causal chain is real and in places already quantitative. The hydroxyproline-to-scurvy sequence and the PIEZO1-to-performance sequence each span many orders of magnitude with support at every link, and establish that the atom-to-cell programme is achievable rather than aspirational.

Second, the mechanically decisive events are coupling properties between structural units rather than properties of the units themselves. Toughness resides in controlled sliding between molecules, fibrils and fascicles and in the cross-link density that permits sliding while resisting flow. Functional specialisation resides in the interfascicular matrix. The principal ageing pathology arises from unregulated cross-linking that suppresses sliding. Single-scale models, which describe units rather than couplings, mislead for this reason.

Third, the hierarchy is a loop rather than a ladder. The tenocyte builds the structure whose mechanics determine the signals it receives, and the matrix protects itself from proteolysis in proportion to load. Tendon homeostasis is a control problem, and the characteristic pathologies dual aetiology of tendinopathy, the AGE ratchet, focal degeneration, and fibrotic healing are loop failures rather than component failures.

Fourth, the mathematics exist but have not been assembled. Every component of a complete multiscale model is available in validated form. What is missing is the downward coupling from cellular output to structural parameters, structural rather than phenomenological parameterisation, and the experimental transfer functions that would constrain it.

Table 9 sets out the research agenda that follows, ordered by our assessment of tractability and consequence. Several developments make the next five years unusually promising. Single-cell and spatial atlases of healthy and diseased human tendons [231,232,233] permit mechanobiological experiments to be designed around identified subpopulations for the first time. Tenocyte-specific channel genetics [39,244] and human variant studies [251] provide the means to determine which sensors are rate-limiting. Atomistic models that now incorporate non-enzymatic cross-links [147] allow the AGE hypothesis to be tested with explicit chemistry. Constitutive model discovery under thermodynamic constraints [317,318] offers a route to tendon models that are both flexible and interpretable. Explant platforms that preserve the pathophysiological niche [226,227] make the closed-loop measurement proposed in Section 7.5 feasible. Finally, the growing recognition that tendon datasets are small and site-confounded [230,232,334] should drive the field toward larger, better-controlled cohorts with explicit null baselines. Framed as a controller of a material, it continuously rebuilds. Operating with slow actuators, an integrating disturbance it cannot reject and a sensor array whose gain it adjusts, the tenocyte presents three therapeutic targets: restoring the stimulus to the homeostatic window, reducing the irreversible chemistry, and, the hardest and most valuable, increasing the turnover rate of a matrix that evolution optimised to last a lifetime without replacement.

## Figures and Tables

**Table 1:** Hierarchical levels of tendon organisation with dominant deformation physics and the modelling formalism conventionally applied at each level.

Level	Characteristic Size	Dominant Deformation Physics	Governing Chemistry	Modelling Formalism	Key Refs.
Residue/bond	0.1–0.9 nm	Backbone dihedral restriction; ring pucker	Hydroxylation; hydrogen bonding	Quantum chemistry; classical force fields	[20,21,26,101]
Tropocollagen	300 × 1.5 nm	Entropic elasticity at low force; enthalpic stretch at high force	Interchain H-bonds; hydration shell	Worm-like chain; all-atom MD	[7,27,102,103]
Microfibril/fibril	4 nm/20–500 nm	Molecular stretch; intermolecular slip; gap/overlap partition	Enzymatic and non-enzymatic cross-links; SLRP binding	Coarse-grained MD; mesoscale bead-spring	[43,67,104,105]
Fibre/fascicle	1–1000 µm	Interfibrillar shear (shear-lag); crimp recruitment	GAG–protein interactions; cross-link density	Shear-lag; recruitment integral; hyperelasticity	[2,31,32,106]
Tendon	1–10 mm	Fascicle sliding; poroelastic flow; bulk viscoelasticity	IFM elastin, lubricin, fibrillin	QLV; HGO; poroelastic FE	[41,82,87,107]
Embedded cell	5–20 µm	Local strain modulation; fluid shear; pressure	Integrin binding; channel gating	Cell-level poroelastic FE; reaction–diffusion	[108,109,110]

MD, molecular dynamics; SLRP, small leucine-rich proteoglycan; GAG, glycosaminoglycan; QLV, quasi-linear viscoelasticity; HGO, Holzapfel–Gasser–Ogden; FE, finite element; IFM, interfascicular matrix.

**Table 3:** Comparative chemistry of enzymatic and non-enzymatic collagen cross-linking.

Feature	Enzymatic (LOX-Mediated)	Non-Enzymatic (Glycation)
Initiating chemistry	Cu^2+^-dependent oxidation of telopeptide Lys/Hyl	Maillard reaction of reducing sugar with helical Lys/Arg
Regulation	Enzymatic; LOX expression is mechanoresponsive [39]	Unregulated; rate ∝ [glucose] × residence time
Location	Telopeptides, defined geometry in the gap region	Helical domain, stochastic
Principal mature species	Hydroxylysyl and lysyl pyridinoline	Glucosepane (most abundant), pentosidine
Effect on toughness	Increases to an optimum, then decreases [104]	Decreases (embrittlement)
Effect on sliding	Permits controlled, recoverable slip	Restricts slip; suppresses dissipation
Reversibility	Remodellable via turnover	Effectively irreversible in a slow-turnover tissue
Human tissue-level evidence	Strong (developmental and inhibitor studies)	Mixed: disorganisation may dominate [148,149]

**Table 4:** Principal non-collagenous constituents of tendon and their established roles.

Component	Class	Localisation	Established Function	Mechanical Role	Refs.
Decorin	SLRP (DS/CS)	Fibril surface	Limits lateral fibril growth; ~80% of PG core protein	Indirect; required in mature tendon	[71,75,76,195]
Biglycan	SLRP (DS/CS)	Pericellular; progenitor niche	Fibrillogenesis; stem-cell niche	Required with decorin	[76,196,197]
Fibromodulin/lumican	SLRP (KS)	Fibril surface	Stage-specific fibril assembly	Null mice: abnormal fibrils, laxity	[178,179,180]
COMP	Thrombospondin	High-load tendon	Catalyses fibrillogenesis	Indirect: assembly rate	[187,188,189]
Tenascin-C	Glycoprotein	Load-bearing regions	Anti-adhesive; mechanoregulated	Modulates cell–matrix coupling	[190,191,192]
Elastin/fibrillin	Elastic fibre	IFM, pericellular	Recoverable extensibility; latent TGF-β store	Governs IFM elasticity and recovery	[72,73,74,193]
Lubricin (PRG4)	Mucinous glycoprotein	IFM, fascicle surfaces	Boundary lubrication	Low-friction fascicle sliding	[86,87,198]
Collagen VI, fibronectin	Pericellular network	Pericellular matrix	Cell–matrix mechanical coupling	Local strain transfer to the cell	[193,199,200]

DS, dermatan sulphate; CS, chondroitin sulphate; KS, keratan sulphate; PG, proteoglycan; COMP, cartilage oligomeric matrix protein.

**Table 5:** Reported elastic moduli across the tendon hierarchy with the principal conditions affecting each value.

Structural Level	Modulus (Reported Range)	Method	Principal Determinants	Refs.
Tropocollagen (enthalpic regime)	3–9 GPa	X-ray diffraction; Brillouin scattering; MD	Hydration; strain rate	[27,44,103,155,156]
Hydrated microfibril	0.2–0.4 GPa (small strain); 0.9–1.4 GPa (>10% strain)	Atomistic MD	Strain-stiffening transition	[43]
Single fibril, hydrated tension	0.4–2.9 GPa	MEMS; AFM tension	Hydration; cross-link maturity	[63,67,105,120,164,167]
Single fibril, dry or indented	5–10 GPa	AFM nanoindentation	Dehydration raises the value ~10-fold	[64,65,66]
Fascicle	0.3–1.0 GPa	Microtensile testing	Gauge length; grip; crimp	[34,86,87,214]
Whole tendon, linear region	0.8–2.0 GPa	Ex vivo; in vivo imaging	Species; site; age; functional class	[12,13,23,210]
Interfascicular matrix (shear)	2–50 MPa	Fascicle pull-out	Age; functional class; elastin	[74,86,87,88]
Ultimate tensile strength	50–150 MPa	Tensile to failure	Species; site; strain rate	[23,83,211]
Failure strain	8–15%	Tensile to failure	Preconditioning; rate	[23,211,212]

**Table 9:** Research agenda: principal discontinuities in the atom-to-cell chain and the experiments or models required to close them.

Priority	Open Question	Why It Matters	Required Approach	Relevant Refs.
1	Constitutive description of the interfascicular matrix	Governs energy-storing function, ageing and rupture; no accepted model exists	Shear and tensile testing across age and functional class; strain-energy formulation with elastin and lubricin terms	[74,86,87,88,207]
2	Subpopulation-resolved mechanobiology	All existing dose–response data average over heterogeneous cells	Fractionation by atlas-derived markers before loading experiments; spatial readouts in explants	[207,231,232,235]
3	Mechanical-to-biochemical transfer functions	No measured relation from strain or shear to Ca^2+^, YAP or transcription in situ	Simultaneous local strain mapping and live signalling readouts in loaded explants	[90,93,109,226]
4	Rate-limiting sensor under physiological load	Determines the input variable of any mechanobiological model	Tenocyte-specific PIEZO1, integrin and cilium manipulation under controlled loading	[39,236,244,251]
5	Fibril length in mature human tendon	Decides between affine and shear-lag load transfer	Volume electron microscopy over millimetre lengths	[33,34,173]
6	AGE versus disorganisation in human failure	Directs intervention in ageing and diabetes	Paired cross-link quantification, structural imaging and mechanics on the same specimens	[96,148,149]
7	Pericellular matrix properties in situ	Cell-level models currently assume them	AFM or optical trapping on cryosections; correlative microscopy	[108,109,223,319]
8	Closed-loop model implementation	Tests whether the mechanostat is stable under physiological loading	Couple turnover equations to structural parameters; global sensitivity analysis	[46,106,311]
9	Senescence in the interfascicular niche	Compartment most affected by ageing; candidate for senotherapy	Senolytic or senomorphic intervention in aged explants with IFM readouts	[207,301,302]
10	Site-balanced human omics with null baselines	Existing datasets are confounded by anatomy	Multi-site biopsies within patients; report anatomy-only baselines	[230,232,334,335]

## Data Availability

No new experimental data were generated in this review. The computational companion described in Section 10, including source code, dataset manifest, configuration files and test suite, is openly available under the MIT licence at https://github.com/lysyloxidase/tenocyte-virtual-cell (development snapshot, accessed on 6 September 2026). The public expression dataset audited in Section 10 is available from the Gene Expression Omnibus under accession GSE26051.

## Abbreviations

AFM	atomic force microscopy
AGE	advanced glycation end-product
CANN	constitutive artificial neural network
COMP	cartilage oligomeric matrix protein
Cx	connexin
DFT	density functional theory
ECM	extracellular matrix
EGR	early growth response
FE	finite element
GAG	glycosaminoglycan
HGO	Holzapfel–Gasser–Ogden
IFM	interfascicular matrix
LOX	lysyl oxidase
MD	molecular dynamics
MMP	matrix metalloproteinase
P4H	prolyl 4-hydroxylase
PPII	polyproline II
QLV	quasi-linear viscoelasticity
SLRP	small leucine-rich proteoglycan
scRNA-seq	single-cell RNA sequencing
TGF-β	transforming growth factor beta
TIMP	tissue inhibitor of metalloproteinases
YAP/TAZ	Yes-associated protein/transcriptional coactivator with PDZ-binding motif
